# Cancer Therapy With TCR-Engineered T Cells: Current Strategies, Challenges, and Prospects

**DOI:** 10.3389/fimmu.2022.835762

**Published:** 2022-03-03

**Authors:** Paul Shafer, Lauren M. Kelly, Valentina Hoyos

**Affiliations:** ^1^ Center for Cell and Gene Therapy, Baylor College of Medicine, Texas Children’s Hospital and Houston Methodist Hospital, Houston, TX, United States; ^2^ Dan L. Duncan Cancer Center, Baylor College of Medicine, Houston, TX, United States; ^3^ Program in Immunology, Baylor College of Medicine, Houston, TX, United States; ^4^ Program in Cancer & Cell Biology, Baylor College of Medicine, Houston, TX, United States

**Keywords:** T cell receptor, TCR, chimeric antigen receptor, CAR, TCR-engineered T cells, TCR T, adoptive cell therapy

## Abstract

To redirect T cells against tumor cells, T cells can be engineered *ex vivo* to express cancer-antigen specific T cell receptors (TCRs), generating products known as TCR-engineered T cells (TCR T). Unlike chimeric antigen receptors (CARs), TCRs recognize HLA-presented peptides derived from proteins of all cellular compartments. The use of TCR T cells for adoptive cellular therapies (ACT) has gained increased attention, especially as efforts to treat solid cancers with ACTs have intensified. In this review, we describe the differing mechanisms of T cell antigen recognition and signal transduction mediated through CARs and TCRs. We describe the classes of cancer antigens recognized by current TCR T therapies and discuss both classical and emerging pre-clinical strategies for antigen-specific TCR discovery, enhancement, and validation. Finally, we review the current landscape of clinical trials for TCR T therapy and discuss what these current results indicate for the development of future engineered TCR approaches.

## Introduction

The past decades have seen rapid advancements in our understanding of the mechanisms underlying the antitumor function of immune cells, and as such adoptive cell therapy (ACT) strategies have emerged as a major platform of cancer therapeutics. A milestone in ACT was the success of tumor infiltrating lymphocyte (TIL) therapy for metastatic melanoma beginning in the 1980s (1). While TIL therapy remains an important ACT modality, the manufacture of TIL products is logistically challenging. ACT efforts have thus largely transitioned towards strategies to engineer peripheral blood T cells with receptors that confer desired antigen specificity. These predominantly include chimeric antigen receptor T cell (CAR T) and T cell receptor engineered T cell (TCR T) therapies. Due to the remarkable efficacy of CAR T therapies in treating B cell malignancies (2), interest in CAR T therapy has eclipsed that of TCR T therapy. However, TCR T therapy is gaining interest as CAR T trials have so far failed to elicit satisfactory responses in the treatment of solid cancers (2), and many believe TCRs may be better suited for the treatment of solid cancers (3). Indeed, exciting clinical results are now emerging that demonstrate safety and efficacy of TCR T therapies in both hematological and solid cancers. In this review we describe the biology of TCRs and tumor antigen targets and discuss state of the art techniques for TCR discovery and preclinical assessment. Finally, we describe the current landscape of TCR T trials and the challenges that remain.

## Biology of TCRs and Tumor Antigen Targets

### Redirecting T Cell Specificity Through Genetic Engineering

**Figure 1:**
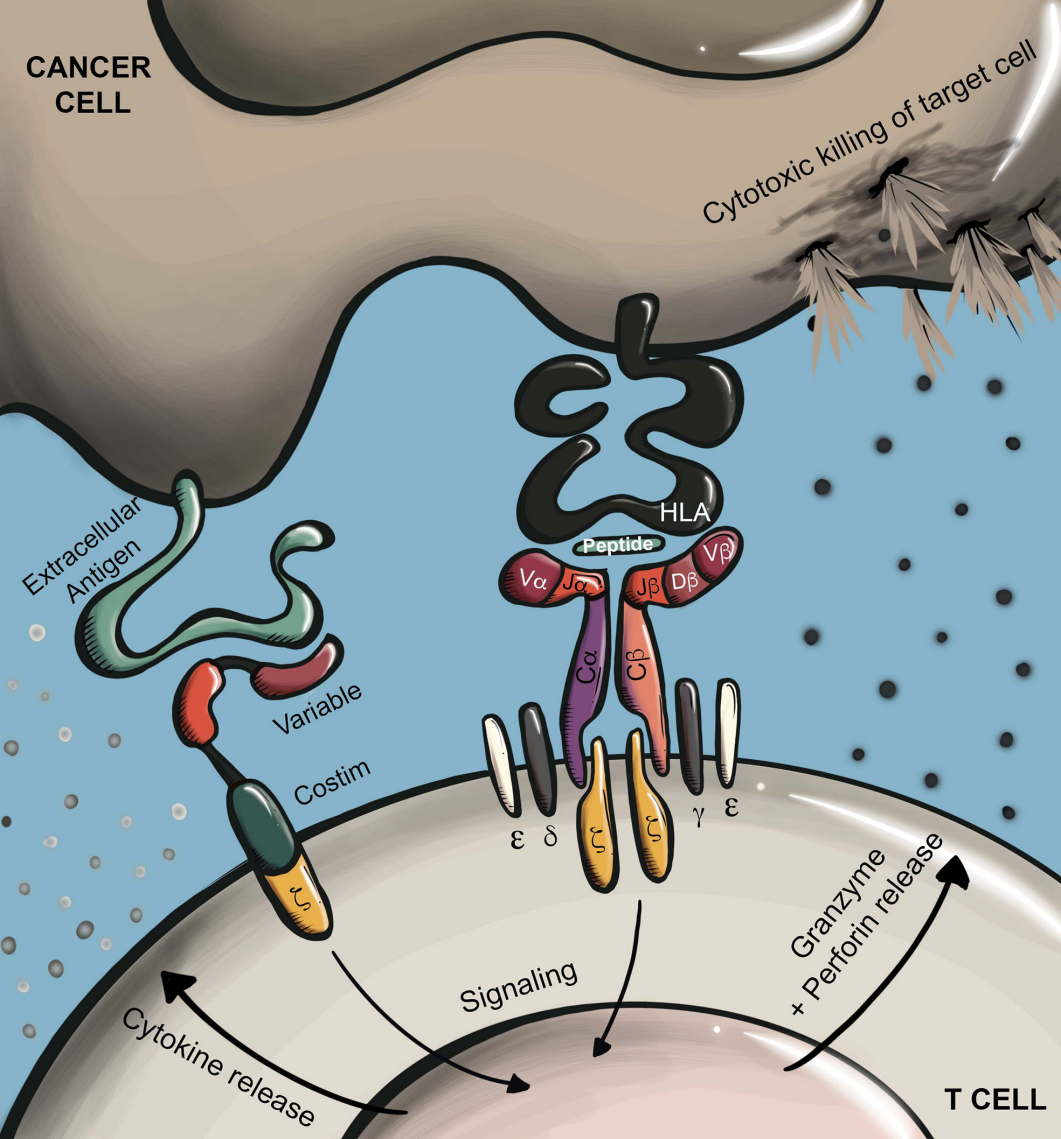
Antigen recognition by CARs and TCRs. CARs recognize surface proteins typically through an antibody-derived scFv recognition domain. Antigen recognition leads to T cell activation *via* phosphorylation of ITAMs in a conjugated intracellular CD3ζ domain. In the case of later generation CARs, ligand binding also leads to additional stimulation of conjugated costimulatory receptors (e.g. CD28, 4-1BB). TCRs recognize HLA-presented peptides which may be derived from any cellular compartment. Antigen recognition by TCRs leads to T cell activation through phosphorylation of ITAMs in the associated CD3ϵ/γ/δ/ζ subunits. Depending on T cell subtype, T cell activation through either receptor type will trigger effector functions including proliferation, cytokine secretion, and target cell killing through directed secretion of perforin and granzyme.

Conventional T cells recognize MHC-presented antigens through their T cell receptor (TCR), a disulfide-linked heterodimer comprised of an α and β chain. To form a functional receptor, TCR α/β heterodimers further complex with CD3ϵ/γ/δ/ζ subunits (4–7). TCRs recognize enzymatically cleaved peptides that are presented at the cell surface by MHC molecules (pMHC). In humans, antigen-presenting MHC alleles are broadly classified as HLA class I (A, B, or C) or HLA class II (DR, DP, or DQ), which predominantly present cytosolic or extracellular derived peptides, respectively (4). The coreceptors CD8 and CD4 enhance TCR antigen sensitivity through interaction with MHC class I or II molecules, respectively (8). TCR binding to cognate pMHC leads to the phosphorylation of immunoreceptor tyrosine-based activation motifs (ITAMs) in intracellular regions of the CD3 subunits (5, 6), which results in T cell activation and initiation of effector functions including proliferation, cytokine secretion, and cytolysis *via* secretion of perforin and granzyme (**Figure 1**). In TCR T therapy, T cells are edited to express TCR α and β chains that confer a desired specificity. Here, introduced TCR α and β chains dimerize and complex with endogenous CD3 components to form a functional TCR that redirects T cell specificity towards an antigen of interest.

Another common method for redirecting T cell specificity is through the genetic transfer of chimeric antigen receptors (CARs), which are broadly comprised of an extracellular-facing antigen-binding domain linked to an intracellular immune cell activation signaling domain. Most often, CARs recognize antigen through the single-chain variable fragment (scFv) of an antibody. In a typical CAR design, the antigen-binding scFv is linked *via* a hinge, or spacer, region to a transmembrane domain that is further conjugated to an intracellular CD3ζ signaling domain. In this manner, antigen binding by the scFv drives CD3ζ phosphorylation and downstream T cell activation. Later generation CARs include the addition of intracellular costimulatory domains such as CD28 and 4-1BB, which further improve CAR T function and persistence (**Figure 1**) (9).

### TCRs vs CARs

While TCRs recognize antigens in the context of HLA presentation, CARs recognize natively folded proteins at the cell surface. Therefore, CARs overcome clinical limitations imposed by the HLA-restriction of TCRs. HLA encoding genes are the most polymorphic in the human genome, with over 20,000 HLA-class I alleles identified to date (10). Therefore, unlike CAR T therapy, patients selected for TCR T therapy must express not only the targeted antigen, but also the corresponding antigen-restricting HLA allele. For this reason, TCR T therapies typically utilize TCRs that are restricted to relatively common HLA alleles, such as HLA-A*02:01, which is present in about 47.8% and 16.8% of Caucasian and African American populations in the United States, respectively (11).

As CARs and TCRs utilize differing signaling mechanisms, they exhibit several important differences in their functional response to antigen stimulation. While TCRs can elicit a cytotoxic response to as few as a single pMHC molecule, CARs typically require thousands of target surface molecules to mediate an effective response (12–14). A consequence of the reduced antigen sensitivity of CARs is seen in patients with B cell malignancies who initially respond to CAR T therapy but subsequently relapse with progression of antigen-low cancer cells (15). Upon stimulation, CARs mediate supraphysiologic T cell activation, leading to enhanced cytokine secretion. For this reason, CAR T cells are more likely to cause cytokine release syndrome (CRS) in patients as compared to TCR T cells; however, recent advancements in treatment have made CRS generally clinically manageable (16, 17).

Tumor antigens recognized by CARs must be located at the surface of cancer cells. Conversely, TCRs recognize HLA presented peptides that may be derived from any cellular compartment. As transmembrane proteins constitute only an estimated 14-26% of the proteome (18–20), CAR-targetable antigens are considerably more limited. However, the repertoire of CAR-targetable antigens is extended to some degree by the ability of CARs to recognize not only protein antigens, but also other molecules like glycoproteins and glycolipids (21). This difference in repertoire of potential targetable antigens has significant implications for CAR T and TCR T therapies, which must aggressively target tumor cells while avoiding toxicity directed towards healthy tissue. Indeed, expression of target antigen on normal cells can lead to T cell-mediated destruction of healthy tissue, known as ‘on-target off-tumor’ toxicity (22). Therefore, the degree to which a target antigen is exclusively expressed by cancer cells is an important factor. So far, the primary success of CAR T therapy has been in the treatment of B cell malignancies targeting CD19, an antigen expressed ubiquitously on malignant and healthy B cells. While CD19-directed CAR T therapy leads to ablation of both malignant and healthy B cells, such on-target off-tumor toxicity is clinically manageable through replacement antibody therapy (22). However, in the case of many other types of cancer, including almost all solid cancers, such T cell-mediated ablation of healthy organ tissue is not clinically manageable, and thus target antigens with exclusivity of expression in cancer cells are best. Currently described tumor antigens with the greatest specificity of expression in cancer cells are predominantly intracellular derived antigens, accessible to TCRs but not CARs (23). Therefore, TCR T therapies may have an advantage over CAR T therapies in the ability to aggressively target cancer cells while minimizing toxicity.

### TCR Targeted Tumor Antigens

**Figure 2:**
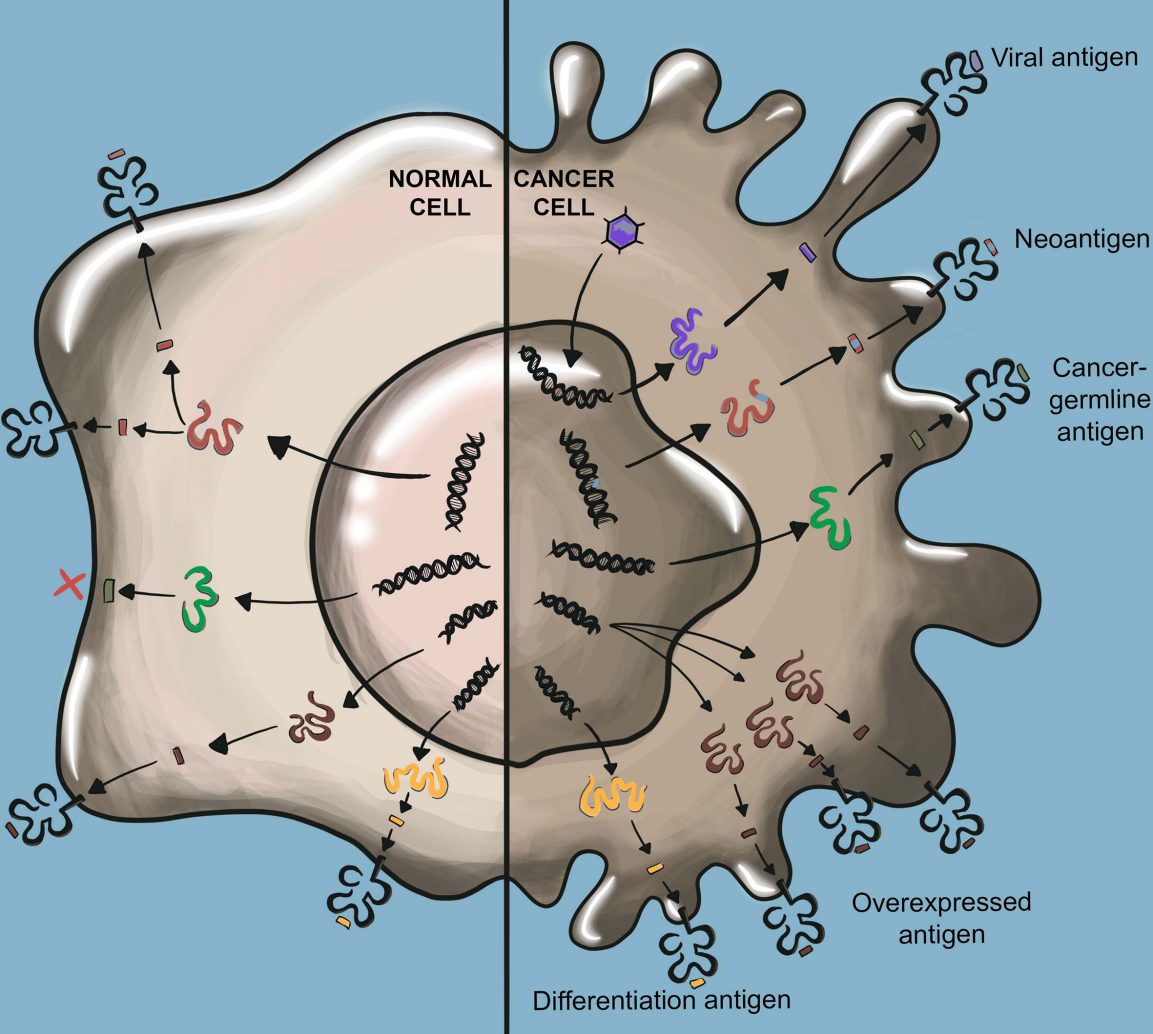
TCR-recognized tumor antigens. Viral antigens result from viral oncogenes which are not present in normal cells. Neoantigens arise from somatic mutations not found in normal cells. Viral antigens and neoantigens are collectively referred to as tumor-specific antigens (TSAs). Cancer germline antigens (CGAs) are derived from proteins that are normally only expressed in germ cells such as testis which lack HLA class I expression. Overexpressed antigens arise from proteins highly overexpressed in cancer tissue as compared to normal tissue. Cancer differentiation antigens are expressed by cancer cells and their expression is otherwise limited to only the normal cells of the same tissue origin as the cancer. Overexpressed antigens and cancer differentiation antigens are collectively referred to as tumor-associated antigens (TAAs).

Significant progress has been made in identifying the precise cancer antigens that mediate immune rejection. While the nomenclature describing tumor antigens varies, widely studied classes of tumor antigens include tumor-associated antigens (TAAs), cancer-germline antigens (CGAs), and tumor-specific antigens (TSAs) (24–26) (**Figure 2**).

#### TAAs

TAAs are expressed by tumor cells but are also expressed in at least some healthy tissue. As a result, therapies targeting TAAs must contend with potential T cell mediated on-target off-tumor toxicity. TAAs are further classified as differentiation antigens or overexpressed antigens.

Differentiation antigens are expressed by cancer cells as well as normal cells of the same tissue origin. Melanoma differentiation antigens were among the first discovered tumor antigens and include the widely studied melanoma-associated antigen recognized by T cells (MART-1) (27) and glycoprotein 100 (gp100) (28). Among tumor antigens, differentiation antigens typically pose the greatest risk for on-target off-tumor toxicity. As discussed in further detail in a later section, clinical experience has demonstrated that targeting differentiation antigens is likely only clinically appropriate when antigen expression is restricted to dispensable healthy tissue, such as CD19-expressing B cells. Likely for this reason, only one TCR T clinical trial targeting a solid cancer differentiation antigen has been initiated since 2012 (**Supplementary Table 1**).

Overexpressed antigens are expressed at high levels in cancer cells but are minimally expressed in healthy cells. While targeting such antigens continues to pose a risk for on-target off-tumor toxicity, the differential expression between cancer and normal cells allows for the possibility of achieving a therapeutic window by which adoptively transferred T cells may destroy high-antigen expressing cancer cells with minimal destruction of low-antigen expressing healthy tissue. An example of a widely studied overexpressed antigen is Wilms’ Tumor Antigen 1 (WT1), a transcription factor with 10- to 1000- fold higher expression in leukemic cells as compared to normal cells (29, 30).

#### CGAs

CGAs are aberrantly expressed in cancer cells while their expression in normal tissue is restricted to germline cells, such as those of testis, which lack HLA-class I expression, thus greatly reducing the risk for on-target off-tumor toxicity. As such, CGAs are currently among the most aggressively pursued targets for TCR-based immunotherapies. Examples of CGAs with high clinical significance include NY-ESO-1 and MAGE-A4, which are detected with high levels of expression in various solid and hematological cancers (31–35). However, several studies have reported that CGAs are heterogeneously expressed within tumors, which could limit potential therapeutic efficacy when targeting a single CGA (36).

#### TSAs

TSAs are genetically encoded in cancer cells but are not present in the genome of any normal cells. TSAs are further classified as viral antigens or neoantigens.

Many human cancers are caused by viral infections, such as human papillomavirus (HPV) (37), hepatitis B virus (HBV) (38), and Epstein-Barr virus (EBV) (39, 40). In many cases, virus-driven cancers are mediated by the expression of viral oncogenes that drive cellular transformation and cancer progression (41–43). As viral oncogenes are often homogenously expressed in virus-driven cancers, and their expression is nearly absent in normal cells, they represent highly attractive tumor antigen targets. Specific examples of clinically relevant viral antigens include the HPV viral oncogenes E6 and E7, which are expressed in several types of epithelial carcinoma (37, 44, 45), and the EBV viral oncogenes LMP1 and LMP2, which are expressed in several solid and hematological cancers (40, 46–49).

Genomic instability is a cardinal feature of cancer (50), which results in the accumulation of many tumor-specific mutations. Some of these mutations will give rise to new proteins, or neoantigens. As neoantigens are expressed exclusively by cancer cells, these serve as attractive targets for ACT that would pose essentially no risk for on-target off-tumor toxicity. However, a challenge is that the vast majority of cancer mutations are so-called bystander mutations, which do not enhance the fitness of the cancer cell. Such random, non-selected mutations are typically heterogeneously expressed and are unlikely to be shared across patients, rendering them ineffective antigen targets. Conversely, a small fraction of cancer mutations improve cellular fitness and directly promote cancer progression, which are known as driver mutations. These mutations may be expressed homogenously by cancer cells and shared among patients within particular cancer types (51–54). If immunogenic and restricted to a common HLA, such driver mutations give rise to so called ‘public neoantigens’ (55–57). While few public neoantigens have been discovered so far, these highly selective targets are of significant clinical interest. Examples of currently described public neoantigens include KRAS G12D/G12V, collectively found in 60-70% of pancreatic adenocarcinomas and 20-30% of colorectal cancers (58), and PIK3CA H1047L, detected in about 5% of metastatic breast cancers (59, 60).

## Strategies for the Isolation and Transgenic Expression of Antigen-Specific TCRs

### Enrichment of Antigen-Specific T Cells

V(D)J recombination of TCRs during thymic development results in a tremendous diversity of TCR sequences within the human T cell repertoire. It is estimated that in an average adult human, there are approximately 4 x 10^11^ total circulating T cells and an estimated 10^10^ unique T cell clonotypes (61). Thus, for the vast majority of T cell clones with specificity towards non-viral antigens, the clonal frequency in peripheral blood is far below what is needed to perform the various manipulations required to isolate antigen-specific TCRs given current technologies. Therefore, TCR isolation efforts generally begin with a method that allows for enrichment of T cells with the desired antigen specificity. The following section describes several common T cell enrichment methods employed for TCR discovery.

#### Expansion of Tumor Infiltrating Lymphocytes

In certain types of solid cancers there is often a large presence of tumor infiltrating lymphocytes (TILs) (62). Compared to peripheral blood T cells, T cells within the tumor tissue are often enriched in clones with tumor-antigen specificity. Several groups have used expanded TILs as sources for discovery of tumor specific TCRs (63–67). Of note, for decades *ex vivo* expanded TILs have in themselves served as an effective ATC for several types of solid cancer (66–70). The TIL therapy lifileucel from Iovance has demonstrated strong efficacy in clinical trials and the company plans to submit for FDA approval (71).

#### Vaccination

T cells with specificity towards antigens of interest can be selectively expanded *in vivo* through vaccination strategies. A common approach is to vaccinate human HLA transgenic mice with an antigen of interest, which can result in robust enrichment of antigen-specific T cells harvested from the lymph nodes and spleen (46, 58, 72). In select cases, the peripheral blood of patients participating in cancer vaccine trials has been used as a source of antigen-enriched T cells for TCR discovery (73, 74).

#### Selective *In Vitro* Expansion of Peripheral Blood T Cells

Several strategies have been developed to stimulate peripheral blood T cells *in vitro* in an antigen-specific manner, driving the selective expansion of T cells with a desired specificity. Early pioneering work in this regard performed *in vitro* stimulations of peripheral blood T cells to preferentially expand virus-specific T cells (75–78). These stimulation methods have since been used to expand T cells enriched in specificity for TAAs (79–81) and neoantigens (60, 82, 83). These approaches typically stimulate T cells *via* autologous antigen presenting cells (APCs), usually dendritic cells (DCs), pulsed with the antigens of interest in the form of exogenous peptide or through cDNA/RNA delivery (60, 80, 82–84). In the case of patient-derived peripheral blood, several studies have shown that initial selection of PD-1+ and/or antigen-experienced (CD45RO+CD62L+, CD45RO+CD62L-, or CD45RO-CD62L-) T cells can further enhance *in vitro* enrichment of tumor-specific T cells (82, 83, 85–87).

To overcome the requirements of generating autologous mature DCs for antigen stimulation, several groups have developed so called artificial antigen presenting cells (aAPCs). One common aAPC system uses the myelogenous leukemia cell line K562, which is negative for HLA-A, B, and DR. This cell line serves as a modular aAPC through the stable transduction of various HLA alleles and costimulatory molecules. Other cell-free aAPC systems have been developed that conjugate HLA and costimulatory molecules onto beads and nanoparticles (88–90).

### Isolation of Antigen-Specific T Cells

After obtaining polyclonal T cell products that are enriched for T cells with specificities of interest, it is necessary to isolate the antigen-specific T cells from the bulk T cell population.

Approaches in this regard typically involve stimulating T cells with the cognate antigen of interest, and then isolating antigen-responsive T cells based on increased expression of known T cell activation-associated molecules. This includes antibody staining of transmembrane proteins that are transiently upregulated following T cell stimulation (e.g., 4-1BB and OX40 in CD8+ and CD4+ T cells, respectively), allowing for isolation of these cells by FAC sorting or magnetic bead separation (82, 83). Another approach is IFN-γ-capture, whereby antigen stimulated T cells are identified and captured based on production of IFN-γ, which is rapidly secreted by antigen-stimulated CD8+ and Th1 CD4+ T cells. In certain cases, staining with peptide-HLA multimers followed by FAC sorting or magnetic bead separation is an efficient method to identify and isolate antigen specific T cells (63). However, this requires upfront knowledge of an antigen restricting HLA and minimal epitope. Although the repertoire of HLA multimer reagents is expanding, these reagents remain limited to relatively common HLA alleles.

### TCR Sequencing

TCR α and β chains of T cells of interest are then cloned from cDNA through PCR amplification. However, a unique challenge is that their 5’ regions are highly variable. To overcome this, one of two PCR variations are typically employed, 5’ RACE or multiplex PCR (66, 67, 73, 79, 82, 91). The fact that TCR specificity is encoded by regions of two separate genes imposes a unique challenge for determining functional TCR sequences from a population of T cells; that is, once T cells are lysed for RNA extraction, the TCR α and β transcripts from each T cell clone intermix, making it ambiguous as to which TCR α sequence pairs with which TCR β sequence. Therefore, prior to sequencing the functional TCR α and β chain transcripts of antigen-specific T cells, it is typically required to first separate individual T cell clones. While by no means exhaustive, we outline here several classical and emerging strategies to isolate T cell clones for TCR sequencing.

#### Limiting Dilution

A classical method for obtaining T cell clones is the outgrowth of T cell clones in individual wells. In the limiting dilution method, T cells are diluted to obtain a cell concentration allowing for approximately one cell to be deposited into each well of a 96-well plate. An alternative method is to FACs sort the T cell population to deliver a single cell into each well. The goal is to obtain expanded clonal populations of the T cells of interest, which can then be additionally screened for antigen-specificity and sequenced *via* Sanger sequencing (27, 46, 72, 73, 84, 92–97).

#### Single Cell RT-PCR

Several studies have obviated the need to expand T cell clones following antigen-specific T cell separation by instead performing single cell RT-PCR to amplify TCR α and β chains. In such methods, single T cells are FAC sorted into wells containing RT-PCR reaction buffer, and from a single cell RT-PCR is performed and the TCR α and β chains are PCR amplified (66, 79, 82, 83, 91, 98). This method reduces the time and labor required for expansion of individual T cell clones; however, a downside to this approach is that confirmatory assays to assess antigen specificity cannot be performed on the T cell clones prior to sequencing.

#### Single-Cell RNA Sequencing

Single-cell RNA sequencing (scRNAseq) is a rapidly advancing technology that has emerged as a uniquely effective platform for TCR discovery, as it allows for single-cell assessment of cellular gene expression as well as the sequence of gene transcripts. As such, several recent studies have successfully used this platform for TCR discovery by stimulating T cells with antigens of interest and then performing scRNAseq. This allowed the researchers to identify antigen specific T cells through their increased expression of effector cytokines such as IFN-γ, TNF-α, and/or IL-2, and from this same data set the researchers then obtained the sequences of transcripts for the TCR α and β chains from the activated cells (60, 65, 99).

## Preclinical Assessment of Candidate TCRs

TCR discovery efforts often yield sequences of several TCRs with a desired antigen specificity. How does one select an optimal TCR from this list of candidates? And once a lead TCR is selected, what preclinical evaluations can be performed that may predict the likelihood of clinical success? The following section describes several TCR features that are commonly evaluated in preclinical TCR studies.

### HLA Restriction

**Figure 3:**
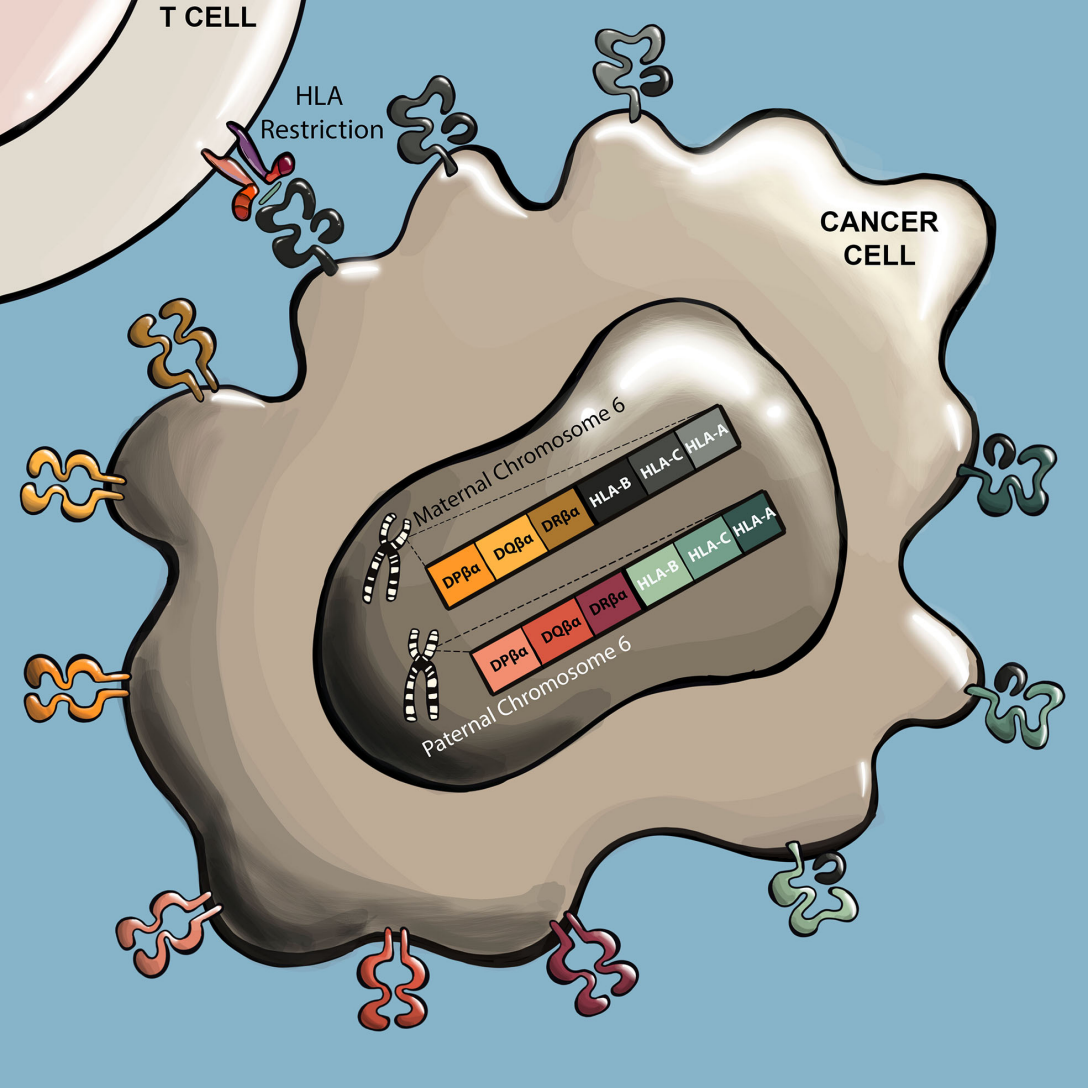
TCRs recognize antigens presented by specific HLA alleles. TCR antigens are predominantly presented by six HLA genes. These include genes for HLA class I (A, B, and C), and class II (DR, DP, and DQ). These HLA genes are highly polymorphic, with many allele variants in the human population. Humans inherit one set of each gene from each parent, and human cells can therefore express up to twelve different HLA presenting alleles. For a given TCR, the specific HLA allele that presents the cognate peptide is referred to as the ‘restricting HLA’ of the TCR.

Often the first characteristic of an isolated TCR that must be determined is its HLA restriction **(** **Figure 3** **).** Not only is understanding the HLA restriction of a TCR necessary to identify patients that may respond to a TCR T therapy, it is also needed to perform many of the experiments for preclinical assessment. In the case of several TCR discovery approaches, knowledge of HLA restriction is already incorporated into the pipeline, such as in the case of vaccination of HLA-transgenic mice or the use of HLA tetramers to select T cells, in which case the HLA restriction of resulting TCRs will be near certain. However, in TCR discovery approaches that do not incorporate *a priori* knowledge of HLA restriction, such as through stimulating with autologous DCs, HLA restriction must be determined experimentally. A commonly used approach is to deliver individual cloned HLA alleles into the non-human primate COS-7 cell line, which possesses antigen processing and presentation capabilities but does not express potentially confounding endogenous human HLA alleles. The COS-7 cells are then induced to express one of the HLA candidates and the antigen of interest through delivery of cDNA/RNA or peptide loading, and cocultured with TCR T cells. Here, the antigen restricting HLA is evident as the HLA that elicits a TCR T cell response, observed through functional responses such as cytokine secretion and/or 4-1BB/OX40 upregulation (58, 66, 82, 83, 87, 93, 100–104).

### TCR Affinity/Avidity

The successful interaction between a TCR and the appropriate pMHC complex is a critical component of effective antitumor immune responses. TCR affinity and avidity describe the binding and kinetic interactions between the TCR and the pMHC (105). Affinity plays a central role in TCR sensitivity and specificity, and refers to the physical strength of the interaction (105–107). Affinity is quantified *via* surface plasmon resonance (SPR), a 3D interaction that measures binding in terms of an association rate (K_on_) and a dissociation rate (K_off_) (105, 107, 108). Together, K_off_ and K_on_ make up the binding constant (K_D_), where K_D_ = k_off_/k_on_ (105, 107). High affinity TCRs recognize lower levels of antigen, do not require the CD8 coreceptor, and can enable CD4+ T cells to recognize and lyse tumor cells in an MHC class I-dependent manner (106, 109).

TCR avidity usually correlates with affinity, and refers to the combined effect of multiple TCR-pMHC interactions, coreceptors (CD8), TCR density, and T cell functional status (105, 110). Different aspects of avidity (i.e. structural or TCR avidity) can be measured *via* staining with pMHC monomers or multimers with defined valency (105). TCR affinity, avidity, and the various kinetic constants all contribute directly/indirectly to functional avidity, which describes how well T cells expressing a specific TCR respond to decreasing abundance of peptide, and is sometimes referred to as antigen sensitivity (105, 108, 111). Assessments of TCR functional avidity typically include measurement of TCR T cell cytokine secretion or cytolytic function in response to target cells that are pulsed with titering concentrations of peptide.

### Antigen Processing by the Standard or Immunoproteasome

HLA class I epitopes are peptide fragments, typically 8-12 amino acids in length (112, 113), generated through processing of ubiquitinated proteins by the proteasome. The proteasome is a large protein complex responsible for the degradation of endogenous proteins that have been damaged or are not needed by the cell and have been tagged by ubiquitin conjugation. The subunits β1, β2, and β5 of the proteasome’s 20S catalytic core are associated with the three major catalytic activities of the proteasome. While proteasomes that incorporate subunits β1, β2, and β5 are referred to as the ‘standard proteasome’, hematopoietic cells and cells stimulated with certain inflammatory cytokines (e.g., INF-γ, IFN-α, IFN-β, and TNF-α) alternatively express β1i, β2i, and β5i subunits that displace β1, β2, and β5 subunits in the proteasome, forming an isoform termed the ‘immunoproteasome’. The immunoproteasome displays several biochemical differences that influence peptide cleavage activity. This results in the immunoproteasome producing peptide products with enhanced immunogenicity compared to the standard proteasome, as these immunoproteasome-generated peptides are more likely to contain C-terminal hydrophobic residues, which are associated with more efficient HLA-class I binding (24, 114). In addition, there are ‘intermediate proteasomes’ that contain a mixture of standard and immunoproteasome subunits, specifically substituting only β5i or β1i plus β5i and result in a peptide repertoire similar to that produced by the immunoproteasome, but includes additional unique peptide products (24, 115).

Several tumor antigens have now been characterized as being produced by the standard, intermediate, and/or immunoproteasomes (101, 115–118). Because the dendritic cells used to enrich tumor specific T cells express predominantly intermediate and immunoproteasomes (115), it is likely that most preclinical TCRs will recognize antigens produced by immunoproteasome and/or intermediate proteasome. These cognate peptides may or may not be additionally produced by the standard proteasome. The proteasomal requirements of a cognate peptide can be determined in several ways. Many cell lines predominantly express the standard proteasome, but will upregulate immunoproteasome subunits in response to IFN-γ. Therefore, the response of TCR T cells can be compared against antigen expressing cell lines with or without pretreatment with IFN-γ (73, 101, 116, 117). Peptide proteasomal requirements have also been determined with further resolution by testing T cell responses against antigen expressing 293 cells with or without overexpression of specific inducible proteasomal β subunits (101, 115–117, 119). There is a growing appreciation of the importance of proteasomal processing dynamics in immunotherapies such as immune checkpoint blockade (120) and TCR T therapy (121). In cases in which a therapeutic TCR recognizes a peptide processed exclusively by the immunoproteasome, it may be useful to select patients whose tumors have confirmed expression of immunoproteasome subunits. As downregulation of immunoproteasome subunits has now been observed in some cancer types (122, 123), it is likely ideal for a therapeutic TCR to recognize an antigen that is generated by both the standard and immunoproteasome.

### Safety Assessment of Lead TCRs

Of major importance is the identification of potential safety concerns of lead TCRs, as previous clinical trials have observed severe cases of both on-target off-tumor toxicities (124–126) and off-target toxicities (127, 128). This section describes state-of-the-art techniques to assess the safety of preclinical TCRs and their cognate-antigen targets.

In cases in which a candidate TCR targets a novel or putative TAA or CGA epitope, it is imperative to preclinically assess its pattern of expression and HLA-presentation in healthy tissue. The importance of such validation was made clear by the fatal neurotoxicity that occurred in two patients following administration of T cells expressing an affinity enhanced TCR recognizing an epitope shared by MAGE-A3 and MAGE-A12. Autopsy performed on these patients revealed infiltration of CD3+CD8+ T cells in the brain. Further investigation identified unexpected expression of MAGE-A12 in a subset of neurons in the human brain (129). Several strategies are now available for preclinical assessment of expression profiles of putative CGA or TAA targets that should be employed. Kunert et al. provide suggested strategies based on their experience of assessing the expression profile of a MAGE-C2 derived epitope that is now being targeted clinically (NCT04729543). As an early step in the assessment of putative TAAs or CGAs, the authors suggest consulting online databases such as The Human Protein Atlas (proteinatlas.org) and the CTdatabase (cta.lncc.br) (130), which compile extensive data throughout literature concerning RNA and protein expression of many genes in both healthy and cancerous tissue (131). An additional tool that has recently emerged for the validation and/or discovery of CGAs and TAAs is the HLA Ligand Atlas (hla-ligand-atlas.org), an open-source, community resource of comprehensive human HLA ligandome data collected originally from 29 distinct non-malignant tissues derived from 21 individuals (113). To experimentally and independently evaluate antigen expression in healthy tissue, Kunert et al. suggest performing qPCR on commercially available cDNA libraries derived from a wide array of healthy tissues types, and if possible, further evaluating protein expression by performing IHC on a panel of healthy tissue types (131).

In addition to establishing the safety of the cognate-antigen target, it is of critical importance to investigate potential off-target reactivities of candidate TCRs. The clinical importance of such investigation was highlighted by the deaths of two patients resulting from off-target reactivity of an affinity-enhanced MAGE-A3-specific TCR towards a Titin-derived epitope expressed in cardiomyocytes (127, 128). Several preclinical strategies are now commonly employed by investigators to identify possible off-target reactivities of candidate TCRs. Several groups have identified specific amino acids within the cognate peptide that are necessary for TCR recognition. This is accomplished by mutating each residue within the cognate peptide and identifying the mutant versions unable to elicit a T cell response. The investigators then searched for all other human peptides containing an identical or similar amino acid motif through the use of webtools such as BLAST and ScanProsite, and then assessed whether these structurally similar peptides elicited a response by the candidate TCR (60, 63, 131–134). In cases in which one or several off-target peptides were identified, the researchers further investigated the immunogenicity of these peptides by determining TCR T cell response at titering concentrations (63, 131, 134) or determining if the off-target peptide is actually capable of being naturally processed (132, 133). While this approach is highly valuable for identifying cross-reactive peptides that are structurally similar to the cognate peptide, it would not identify structurally dissimilar peptides that mediate cross-reactivity (135).

Several groups have also assessed potential alloreactivity of therapeutic TCRs by performing functional assays in which TCR T cells are cultured with many different lymphoblastoid cell lines (LCLs) expressing various HLA alleles (131–133). The utility of this approach is highlighted by Sanderson et al., who identified that a lead HLA-A*02:01 restricted MAGE-A4 specific TCR mediated an alloresponse to HLA-A*02:05, indicating that patients that express HLA-A*02:05 should be excluded from treatments using this TCR (133).

## Engineering Strategies to Improve TCR Safety and Efficacy

### Promoting Proper Pairing of TCR α/β Chains

**Figure 4:**
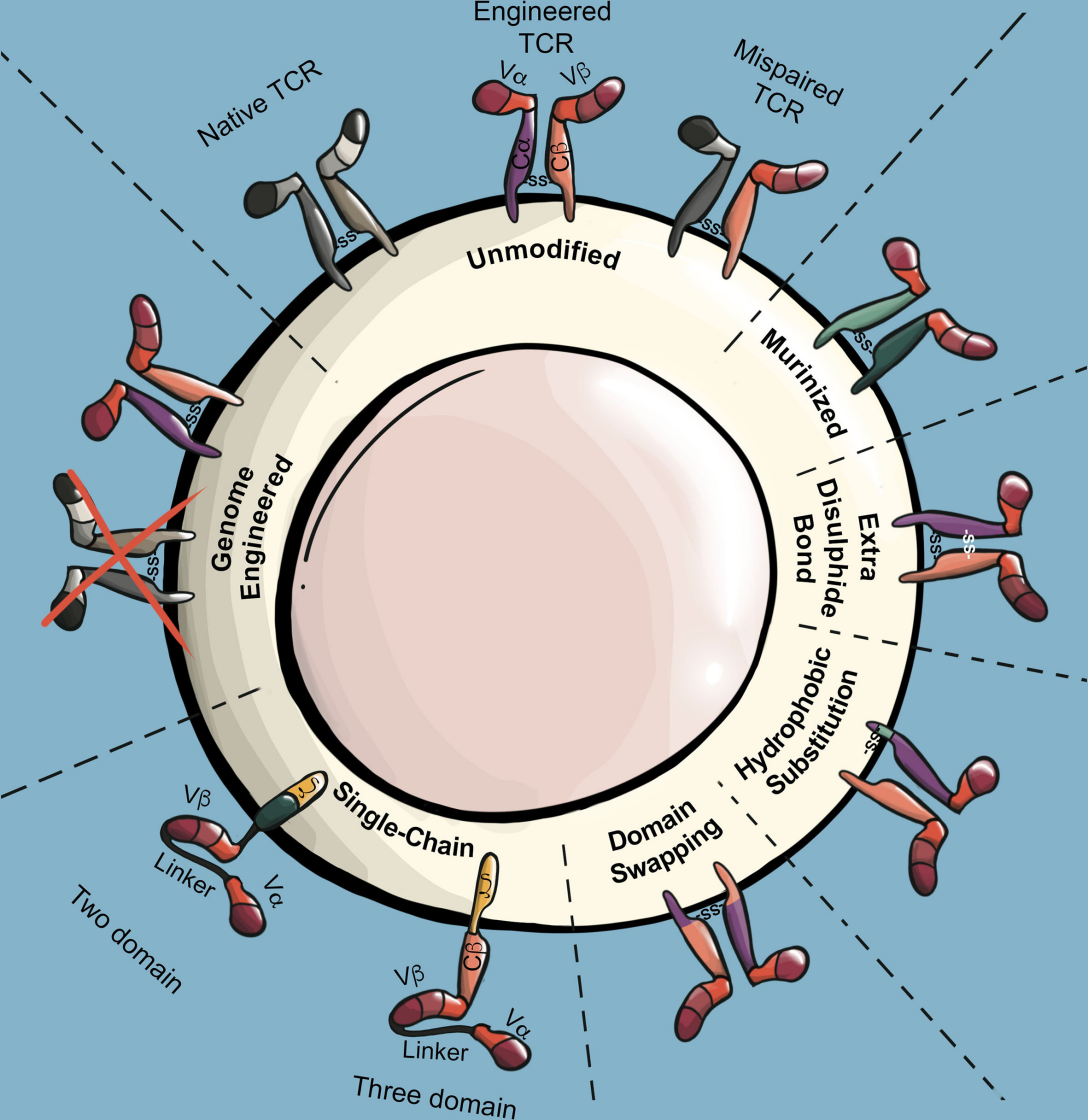
TCR modifications to prevent mispairing and maximize surface expression. Illustration of mispairing between endogenous TCR and engineered TCR. Murinized TCRs replace the human TCR constant regions with those of a mouse TCR constant region. The addition of an extra disulfide bond in the TCR constant region through cysteine substitutions stabilizes interchain binding affinity of engineered TCR α/β chains while reducing their binding affinity with endogenous TCR α/β chains. Stability of the engineered TCR α chain can be increased through select hydrophobic substitutions in its transmembrane region. Domain swapped TCRs invert large or specific segments of the engineered TCR α/β constant regions, which reduces propensity of engineered TCRs to mispair. Single-chain TCRs (scTCR) encode TCR antigen recognition and signaling domains into a single chain. Three-domain and two-domain scTCRs differ by the inclusion or absence of the TCR β constant region, respectively. Genome engineering strategies utilize RNA interference or endonuclease technologies to reduce or ablate endogenous TCR expression.

TCR α/β chains form heterodimers largely through interactions within TCR constant regions. A challenge facing TCR T applications is that the endogenous TCR α/β chains expressed by conventional T cells can pair with introduced TCR α/β chains. Several consequences arise from such TCR mispairing. Firstly, TCR mispairing reduces the surface expression of introduced TCRs, as a significant fraction of the introduced TCR α/β chains will participate in non-productive mispairings with endogenous TCR α/β chains. Furthermore, mispaired TCRs compete with the engineered TCR heterodimer for association with limiting CD3 components (136). A second consequence of TCR mispairing is the production of brand new TCRs that have not undergone thymic selection, and which may have unexpected specificity for autoantigens. Indeed, TCR mispairing was shown to cause lethal graft-versus-host-disease (GVHD) in mice (137), and led to the formation of alloreactive and autoreactive human T cells *in vitro* (138). However, incidence of GVHD has not been observed in human TCR T clinical trials to date, including early TCR T trials utilizing unmodified human TCRs (139). To avoid issues associated with TCR mispairing, the vast majority of TCR T applications now use at least one strategy to reduce TCR mispairing (**Figure 4**).

#### Murinization

Extending on the serendipitous observation that human T cells exhibited greater biological activity when engineered with a murine-derived TCR as compared to human-derived TCRs, Cohen et al. demonstrated that murine TCR α/β chains preferentially dimerize with each other in the presence of endogenous human TCR α/β chains. The investigators further demonstrated that preferential pairing of introduced TCRs is also achieved when the constant regions of human TCRs are replaced with murine constant regions (140). A concern of using engineered TCRs with murine constant regions is that the foreign murine sequences may elicit an immune response in patients, as has been observed in other cell therapy trials utilizing foreign proteins such as green fluorescent protein (141, 142) and the HyTK suicide gene (143). One study identified anti-murine TCR antibodies in the post-treatment sera of 6/26 patients treated with TCR T cells expressing fully murine TCRs. However, epitope mapping revealed that the antibodies were specific for the variable regions of the TCRs, not the constant regions (144). In a separate TCR T trail utilizing a human TCR with murinized constant regions, anti-TCR serum antibodies were not detected in any of the 11 patients screened (44). Together, these clinical findings suggest that murine TCR constant regions have low or negligible immunogenicity. Nonetheless, strategies have also been developed to partially murinize TCRs by substituting specific murine amino acid sequences (145, 146).

#### Additional Disulfide Bond

Endogenous TCR α/β chains form a disulfide bond between TCR α constant region (Cα) residue 94 and TCR β constant region (Cβ) residue 130 (147). The proper pairing of introduced TCRs can be improved by introducing a second stabilizing disulfide bond through cysteine substitutions at Cα residue 48 and Cβ residue 57, which increases interchain binding affinity of introduced TCR α/β chains while decreasing binding affinity with endogenous TCR α/β chains (148, 149).

#### Transmembrane Hydrophobic Substitutions

The endogenous TCR α chain has a relatively low stability, which can be increased by substituting leucine and valine residues within the Cα transmembrane region. TCR α chains containing these stabilizing mutations, termed α-LVL, demonstrate increased TCR surface expression and biological activity. While this strategy promotes pairing of an introduced TCR by stabilizing the TCR α chain, the TCR β chain remains unmodified and thus susceptible to mispairing. However, this can be addressed by incorporating the α-LVL substitutions into murinized TCRs, the combination of which can synergistically enhance TCR expression and biological activity (150).

#### Domain Swapping/Conjugation

Through TCR crystal structure analysis, Voss et al. identified several amino acids mediating TCR α/β dimerization. By swapping two such interacting residues, a Cα glycine and Cβ arginine, the authors generated mutant TCR α/β chains with a similar propensity for dimerizing with each other, but with a significantly reduced propensity to bind with unmodified endogenous TCR α/β chains (151). Bethune et al. employed a similar strategy when they designed TCRs with large regions of Cα and Cβ segments exchanged, referred to as dsTCR_c_. Interestingly, mispairing of introduced dsTCR_c_ α/β chains with endogenous α/β chains was completely undetectable (152). Other examples of TCR domain swapping/conjugation strategies include swapping with γδ TCR constant regions (152), replacing regions with CD3ζ (153, 154) or CD28/CD3ϵ (155), or conjugation to leucine zipper dimerization motifs (156, 157).

#### Single-Chain TCRs

To combine the antigen recognition properties of a TCR α/β heterodimer into a single chain, several groups have developed so-called three-domain single-chain TCRs (scTCR), which are composed of Vα/Vβ regions fused by a short peptide linker and conjugated to a Cβ domain (153, 158, 159). To mediate signal transduction, three-domain scTCRs are typically further conjugated to CD3ζ (153, 158, 160). Zhang et al. compared the function of nine three-domain scTCR constructs conjugated to CD3ζ with or without conjugation to the additional stimulatory domains CD28 and Lck. Although the addition of both CD28 and Lck improved scTCR function, none of the scTCR constructs performed as well as native TCRs in terms of functional avidity (160). scTCR constructs utilizing CD3ζ transmembrane and signaling domains function independently of the CD3 complex (160), which theoretically allows for higher surface expression to be achieved with scTCRs than with native TCRs, as scTCRs are not limited by the abundance of CD3 components. The CD3-independence of scTCRs may also be beneficial in applications where it is desirable to maintain levels of endogenous TCR expression. However, scTCRs utilize signaling mechanisms distinct from those of CD3-dependent native TCRs, which may partially explain the reduced functional avidity of scTCRs (160). To generate scTCRs that preserve CD3-dependence, Voss et al. designed a system whereby three-domain TCRs without CD3ζ conjugation were coexpressed with a Cα domain. In this manner, the three domain scTCR dimerizes with the coexpressed Cα domain, presenting at the cell surface in a four-domain structure similar to that of a native TCR heterodimer. Intriguingly, these scTCR/Cα constructs have similar functional avidities as native TCRs (161). However, Aggen et al. demonstrated that three-domain scTCRs continue to mispair to some extent with endogenous TCR α chains due to the presence of the Cβ domain. To generate a scTCR system that completely eliminates mispairing, the group generated two-domain scTCRs, which utilize stabilizing Vα/Vβ mutations to obviate the need for a Cβ domain (162). To mediate signaling, two-domain scTCRs are conjugated to intracellular signaling domains such as CD3ζ and CD28. Intriguingly, CD3ζ/CD28 containing two-domain scTCRs are essentially CARs that utilize a Vα/Vβ antigen recognition domain. As such, two-domain scTCRs display features typical of CARs including CD3-independent signaling and decreased sensitivity to low antigen density. However, unlike typical CARs, two-domain scTCRs are still dependent on HLA presentation (14).

#### Genome Engineering Strategies

Rather than modifying the introduced TCR, other strategies address mispairing through knock-down or knock-out of the endogenous TCR. Provasi et al. combined the use of zinc-finger nucleases (ZFN) to knock-out endogenous *TRAC* and *TRBC* genes with lenti-viral delivery of a WT-1 specific TCR. Here, the authors described an elegant, although relatively extensive manufacture system utilizing sequential rounds of *TRAC/TRBC* disruption, magnetic bead separation, and TCR α/β chain delivery. This resulted in a TCR T product with enhanced expression of the introduced TCR and a complete absence of endogenous TCR α/β chains (163). In a recent first-in-human trial, Stadtmauer et al. employed multiplexed CRISPR/Cas9 editing to disrupt T cell *TRAC*, *TRBC*, and *PDCD1* genes in combination with lenti-viral delivery of a NY-ESO-1 specific TCR. In the four patient-derived products described in this report, disruption of *TRAC* and *TRBC* was achieved in an average of 45% and 15% of cells, respectively. However, as *TRAC*/*TRBC* edited T cells were not selected prior to lenti-viral transduction with the NY-ESO-1 TCR, a significant fraction of the TCR-engineered T cells likely continued to express endogenous TCR α/β chains (164). Several groups have also developed virus-free systems to deliver TCRs and/or disrupt endogenous *TRAC*/*TRBC* genes, which may also aid in improving TCR T clinical cost and feasibility. Davo et al. electroporated T cells with Dicer-substrate small interfering RNAs (DsiRNA) targeting the endogenous *TRAC* and *TRBC* loci, achieving an approximately 6-fold and 3-fold reduction in expression of *TRAC* and *TRBC*, respectively. The authors then electroporated the T cells with a codon optimized WT1-specific TCR that isn’t recognized by the DsiRNA. This resulted in T cell products with relatively high engineered TCR expression (60.2% tetramer+) with no observable TCR mispairing. However, as transgene expression in this system is transient, this would likely necessitate multiple infusions in a clinical setting (165). Roth et al. used CRISPR/Cas9 editing to mediate targeted insertion of TCR α and β variable regions into the first exon of the *TRAC* and *TRBC* loci, respectively. This mediated the combined effect of disrupting the endogenous TCR α/β chains, while placing expression of the introduced TCR under physiologic control. A potential challenge of this approach is the relatively low editing efficiency, with about 3% of cells expressing the introduced TCR, which could therefore necessitate sorting and/or extended selective expansion (166).

### Affinity/Avidity Enhancement

Given that affinity plays a central role in TCR function, the manufacturing of high-affinity TCRs is an attractive method to improve the efficacy of TCR T therapies. Naturally occurring TCRs, including those that recognize self/tumor antigens, have relatively low affinities as a result of negative selection (105, 167). Methods to improve TCR affinity focus on the introduction of amino acid sequence (AAS) variations into the TCR complementarity-determining regions (CDRs). For example, single and dual AAS substitutions that enhanced the functions of TCRs specific for NY-ESO-1 (1G4) and MART-1 (DMF4, DMF5) were generated through overlapping PCR (168). These TCRs had affinities in the low μM and even nM range, which surpass the affinity of most naturally occurring TCRs, which range from 1-100 μM (106, 110). Yeast and bacteriophage display are additional powerful, high-throughput tools that can generate TCRs with affinities in the pM range (109, 167, 169). While these techniques are effective at identifying high affinity TCR variants, higher affinity has been associated with increased cross-reactivity (106, 109, 168). TCRs with affinities greater than the normal range (1-100 μM) are more likely to demonstrate cross-reactivity to similar or completely different peptides (109, 135, 170). In various studies of high affinity TCRs, increasing the affinity within the nM and pM range resulted in recognition of control antigen and antigen negative target cells (109, 168). Efforts to improve the affinity of TCRs should thus proceed with caution and thorough evaluation as these high affinity TCRs could have detrimental effects when used as patient therapies.

Recent work in TCR affinity maturation has focused on incorporating a more thorough assessment of the structure of the TCR and how it interacts with the target pMHC. Hellman et al. utilized a structure-guided design that incorporated both positive and negative designs (106, 170). In other words, they utilized mutations that either enhanced or weakened the interaction of the TCR with the MHC protein. These mutations redistributed the binding free energy in a way that forces the TCR-pMHC interaction to rely more on the presence of the correct target peptide, leaving less flexibility for off-target peptides. In the MART-1 specific DMF5 TCR, these structure-guided modifications decreased cross-reactivity to MART-1 homologs and eliminated cross-recognition of a selection of divergent peptides. Structure-guided approaches, therefore, have the potential to improve ACT while minimizing the risk of off-target toxicities.

## Clinical Landscape of TCR T Therapies

### Trends in TCR T Trials

**Figure 5:**
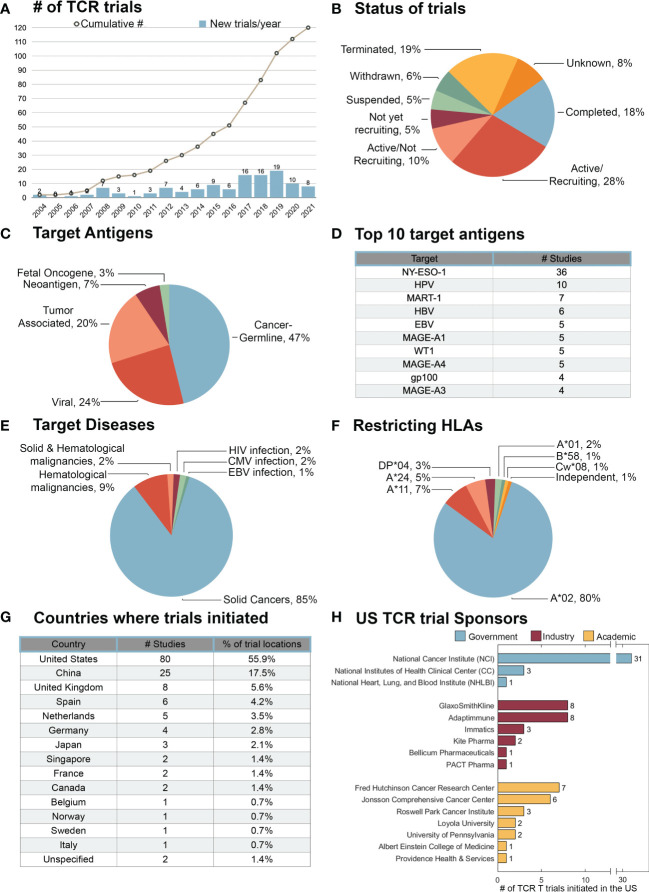
Trends in TCR T trials initiated thus far. TCR T trials registered in clicaltrials.gov were assessed as of October 3^rd^ 2021. **(A)** The number of new TCR T trials initiated each year and the cumulative number of registered TCR T trials by year. **(B)** Clinical status of the 120 TCR T trials. **(C)** Classifications of 118 tumor antigen targets in 116 TCR T trials with specified target antigens. **(D)** Ten most common targets in TCR T trials. **(E)** Diseases targeted in TCR T trials. **(F)** Frequency of 111 target antigen-restricting HLAs in 100 TCR T trials that specified HLA restriction. **(G)** Locations where TCR T trials have been conducted by country. **(H)** Primary sponsors of the 80 TCR T trials conducted in the United States.

As of October 3^rd,^ 2021, the search term “TCR” (and synonyms “T Cell Receptor” and “T Cell Antigen Receptor”) in clinicaltrials.gov yielded 538 interventional trials. Through manual inspection of these trials, 119 were identified to include the adoptive transfer of TCR T cells. One TCR T trial that did not include these search terms (NCT04044768) was also identified and included in this analysis (**Supplementary Table 1**). The first TCR T trial was initiated in 2004 by Steven Rosenberg at the National Institutes of Health (NIH) targeting the melanoma differentiation antigen gp100 (NCT00085462). Since then, the number of new TCR T trials initiated has steadily increased, with a particular acceleration between the years of 2017 – 2019, in which 51 new TCR T trials were initiated (**Figure 5A**). Of the TCR T trials to-date, 53% have been in phase I, 24% in phase I/II, and 22% in phase II. To date, no phase III TCR T trials have been initiated (**Supplementary Table 1**). The status of these TCR T trials was assessed as of November 3^rd^, 2021 (**Figure 5B**). There were 118 antigens targeted in the 116 TCR T trials with specified targets. Of the targeted antigens, the majority are CGAs (47%), followed by viral antigens (24%), tumor-associated antigens (21%), neoantigens (7%), and fetal oncogenes (3%) (**Figure 5C**). The CGA NY-ESO-1 is by far the most targeted antigen in TCR T trials to-date (36 trials). Although not first targeted until 2014, HPV now constitutes the second most common TCR T target (10 trials). While the melanoma differentiation antigen MART-1 is the third most targeted antigen in TCR T trials (7 trials), these largely constitute early TCR T trials, as MART-1 targeted-trials have not been initiated since 2012 (**Figure 5D**). The majority of the TCR T trials to date have been for the treatment of solid cancers (85%), followed by hematological malignancies (9%), and trials targeting both solid and hematological cancers (2%). A small subset of TCR T trials have been for the treatment of HIV, CMV, or EBV infections (4%) (**Figure 5E**). More information about the precise disease targets of these TCR T trials can be found in **Supplementary Table 1**. Of the 100 trials with specified HLA-restrictions, 111 restricting-HLA alleles were listed, as some trials included multiple antigen targets and HLA restrictions. HLA-A*02 was by far the most common restricting HLA allele (80%), followed by HLA-A*11 (7%) and HLA-A*24 (5%). HLA class II restricted antigens were targeted in 3 trials, all of which were restricted by HLA-DP*04 (3%) (**Figure 5F**). The majority of TCR T trial locations were in the United States (56%), followed by China (18%), and the United Kingdom (6%) (**Figure 5G**). Among the 80 TCR T trials occurring in the United States, 44% were sponsored by the NIH, 28% by academic institutions, and 29% by industry. The support of industry in TCR T, which accelerated around 2017 (**Supplementary Table 1**), will likely aid in the development of later phase TCR T trials. The NCI has been by far the most active individual institution in United States TCR T trials, sponsoring 31 trials to date. Among academic institutions, the Fred Hutchingson Cancer Research Center has sponsored the most trials (7 trials), followed by the Johnson Comprehensive Cancer Center (6 trials). Among TCR T trials sponsored by pharmaceutical companies, Adaptimmune and GlaxoSmithKline have sponsored the most trials (8 trials each) (**Figure 5H**).

### Assessment of Safety and Efficacy of TCR T Trials to Date

There is a quickly expanding body of literature detailing the results of TCR T trials. Clinical results encompassing over twenty-five TCR T trials are detailed in **Table 1**. This section discusses broad findings that have emerged from these early phase trials, particularly relating to TCR T safety and efficacy. Of note, many TCR T clinical protocols include lymphodepleting regimens prior to TCR T infusion, which has been demonstrated in early ACT trials to improve T cell engraftment and persistence. Many studies also include systemic administration of IL-2 following TCR T infusion to support T cell activity and persistence. Both interventions consistently induce various toxicities that, while undesirable, are generally clinically manageable. Detailed description of the impact of these interventions is beyond the scope of this review, but is extensively reviewed elsewhere (189–191). As such, discussion of toxicities observed in TCR T trials will focus on those mediated directly by the infused T cells. Finally, it is worth noting that to date all TCR T trials have been early phase and almost exclusively treating patients with highly advanced, treatment refractory disease.

**Table 1:** Available TCR T trial results.

Ref	Year	Trial	Phase	Sponsor	Target	HLA	Construct Details	Disease	# Pts	Pre. Cond.	Combination	Responses	TCR T Induced Toxicity
(124)	2011	NCT00923806	I/II	NCI	CEA	A*02:01	affinity enhanced TCR	colorectal cancer	3 pts	Cy + Flu	IL-2	**1/3, 33.3% (ORR/PR)**	Severe colitis (3 pts)
(126)	2009	NCT00509496	II	NCI	gp100	A*02:01		melanoma	16 pts	Cy + Flu	IL-2	**3/16, 18.8% (ORR)** 1/16 (CR), 2/16 (PR)	Skin toxicity (15 pts), Uveitis (4 pts), Hearing loss (5 pts)
(171)	2006	Not Specified (NS)	NS	NS	MART-1	A*02:01	DMF4 TCR	melanoma	17 pts	Cy + Flu	IL-2 Peptide vaccine	**2/17, 12% (ORR/PR)** 1/17 (MR)	None
(126)	2009	NCT00509288	II	NCI	MART-1	A*02:01	DMF5 TCR	melanoma	20 pts	Cy + Flu	IL-2	**6/20, 30% (ORR/PR)**	Skin toxicity (14 pts), Uveitis (12 pts), Hearing loss (10 pts)
(172)	2014	NCT00910650	II	JCCC	MART-1	A*02:01	DMF5 TCR	melanoma	10 pts	Cy + Flu	IL-2 Peptide-pulsed DC vaccine	**0/14, 0% (ORR)** 7/14 (SD) *ORR assessed at Day 90*	Erythematous skin rash (3 pts), Acute respiratory distress (2 pts)
(173)	2018	NCT01586403	I	Loyola University	tyrosinase	A*02	TIL1383I TCR	melanoma	3 pts	Cy + Flu	IL-2	**1/3, 33% (ORR/PR)**	Vitiligo (2 pts)
(174)	2015	NCT00670748	II	NCI	NY-ESO-1	A*02:01	1G4-a95:LY TCR (affinity enhanced)	melanoma synovial sarcoma	38 pts	Cy + Flu	IL-2 ± AVIPOX- ESO vaccine	**22/38, 58% (ORR)** 5/38 CR, 17/38 PR	None
(175)	2019	NCT02366546	I	Mie University	NY-ESO-1	A*02:01 A*02:06	affinity enhanced TCR siRNA *TRAC/TRBC*	various solid tumors	9 pts	Cy	None	**3/9, 33.3% (ORR/PR)**	CRS (3 pts)
(176)	2019	NCT02070406 NCT01697527	I	JCCC	NY-ESO-1	A*02:01	1G4-a95:LY TCR (affinity enhanced)	various solid tumors	10 pts	Cy + Flu	IL-2, DC-peptide vaccine, ± ipilimumab	**2/10, 20% (ORR)** 1/10 (CR), 1/10 (PR), *4/10 (tR)*	None
(177)	2019	NCT02869217	Ib	University Health Network	NY-ESO-1	A*02:01 A*02:06	TBII-1301 (MS3II-NY-ESO1-SiTCR)	various solid tumors	9 pts	Cy	None	**2/9, 22.2% (ORR/PR)** 5/9 (SD), *1 pending*	CRS grade 1-2 (5 pts)
(178)	2019	NCT01352286	I/IIa	GlaxoSmithKline	NY-ESO-1/LAGE-1	A*02:01	NY-ESO-1 SPEAR T cells (NY-ESOc259 TCR)	multiple myeloma (Post-HSCT)	25 pts	melphalan	lenalidomide	**21/25, 84% (ORR)** 2/25 (sCR), 1/25 (CR), 13/25 (VGPR), 5/25 (PR), 4/25 (SD)	GVHD (6 pts). *Likely not related to the engineered TCR.*
(179)	2019	NCT01343043	I/II	GlaxoSmithKline	NY-ESO-1	A*02	NY-ESO-1 SPEAR T cells	synovial sarcoma	42 pts	Cy ± Flu as per cohort	None	**15/42, 35.7% (ORR)** 1/42 (CR), 14/42 (PR), 24/42 (SD)	CRS grades 1 (2 pts), 2 (1 pts), and 3 (2 pts)
(164)	2020	NCT03399448	I	University of Pennsylvania	NY-ESO-1	A*02:01	8F TCR, CRISPR KO *TRAC*/*TRBC*/*PDCD1*	multiple myeloma, MRCLS	3 pts	Cy + Flu	None	**0/3, 0% (ORR)** 2/3 (SD)	None
(128)	2013	NCT01350401 NCT01352286	I/II	Adaptimmune GlaxoSmithKline	MAGE-A3	A*01	MAGE-A3a3a TCR (affinity enhanced)	melanoma, myeloma (Post-ASCT)	2 pts	Cy (melanoma pts)	None	NA	Off-target toxicity in cardiac tissue leading to 2 pt deaths
(129)	2013	NCT01273181	I/II	NCI	MAGE-A3	A*02:01	affinity enhanced TCR	various solid tumors	9 pts	Cy + Flu	IL-2	**5/9, 55.6% (ORR)** 1/9 (CR), 4/9 (PR)	Neurological toxicity (3 pts) 2 pt deaths.
(194)	2017	NCT02111850	I/II	NCI	MAGE-A3	DPB1*04:01		various solid tumors	17 pts	Cy + Flu	IL-2	**4/17, 23.5% (ORR)** 1/17 (CR), 3/17 (PR)	Fever (10 pts), Elevated ALT, AST, and creatinine (2 pts)
(181)	2015	UMIN000002395	I	Mie University	MAGE-A4	A*24:02		esophageal cancer	10 pts	None	Peptide vaccine	**0/10, 0% (ORR/PR)** 3/10 SD	None
(182)	2020	NCT03132922	I	Adaptimmune	MAGE-A4	A*02	ADP-A2M4 SPEAR T cells	various solid tumors	34 pts	Cy + Flu	None	**7/28, 25% (ORR/PR)** 11/28 (SD)	2 pt related deaths (aplastic anemia and CVA), not likely off target toxicity
(183)	2020	NCT04044859	I	Adaptimmune	MAGE-A4	A*02	ADP-A2M4CD8 SPEAR T cells	various solid tumors	5 pts	Cy + Flu	None	**2/5, 40% (ORR/PR)** 3/5 (SD)	No DLTs or SAEs
(184)	2021	NCT04044768	II	Adaptimmune	MAGE-A4	A*02	ADP-A2M4 SPEAR T cells	synovial sarcoma, MRCLS	37 pts	Cy + Flu	None	**13/33, 39% (ORR)** 11/33 (PR), 2/33 (CR), 15/33 (SD)	CRS grades 1-2 (21 pts), 3 (1 pt)
(185)	2018	NCT02989064 NCT02592577	I	Adaptimmune	MAGE-A10	A*02	MAGE-A10c796 TCR (affinity enhanced)	various solid tumors	8 pts	Cy ± Flu	None	**0/8, 0% (ORR/PR)**	CRS (1 pt), Increase in serum amylase (1 pt)
(186)	2019	NCT02280811	I/II	NCI	E6	A*02:01	E6 TCR	HPV-associated solid cancers	12 pts	Cy + Flu	IL-2	**2/12, 16.7% (ORR/PR)** 4/12 (SD)	None
(44)	2021	NCT02858310	I	NCI	E7	A*02:01	E7 TCR	HPV-associated carcinomas	12 pts	Cy + Flu	IL-2	**6/12, 50% (ORR/PR)** 4/12 (SD)	1 DLT, not likely off target toxicity
(187)	2017	UMIN000011519	I	Several sponsors	WT1	A*24:02	TAK-1 TCR, siTCR	AML, MDS	8 pts	None	Peptide vaccine	Transient decrease of blasts in BM in 2 pts. SD in 1 pt.	No adverse events greater than grade 3
(188)	2019	NCT01640301	I/II	Fred Hutchinson CRC	WT1	A*02:01	TCR_C4_, Allo EBV-specific T cells	AML (Post-HCT)	12 pts	None	IL-2	100% RFS vs 54% in comparative group	GVHD in several patients, not likely caused by TCR T cells, but rather HCT.

NA, Not Applicable. Bolded values highlight the overall response rates of the trials.

#### Trials Targeting Cancer Differentiation Antigens: Evidence of Efficacy and On-Target Off-Tumor Toxicity

In one of the earliest TCR T trials, patients with metastatic melanoma were treated with autologous T cells transduced with a TCR (DMF4) recognizing the melanoma differentiation antigen MART-1. The objective response rate in these patients was relatively modest, with 2/17 (12%) patients achieving durable partial responses. No TCR T induced toxicity was observed (171). In a later related study, patients were treated with TCR T cells that expressed a higher affinity MART-1 specific TCR (DMF5). Compared to the prior trial using the lower affinity DMF4 receptor, this trial observed enhanced efficacy, with objective responses observed in 6/20 (30%) patients. However, the increased biological activity mediated by the DMF5 TCR was also associated with the emergence of on-target off-tumor destruction of melanocytes, leading to widespread erythematous skin rash (14 pts), uveitis (12 pts), and hearing loss (10 pts). Similar results were observed in patients treated with T cells expressing a high affinity mouse-derived TCR targeting the melanoma differentiation antigen gp100 (126). In a later study, three patients with metastatic colorectal cancer were treated with an affinity-enhanced TCR recognizing the cancer differentiation antigen carcinoembryonic antigen (CEA). While a partial response was observed in 1/3 (33%) patients, all three patients presented with severe transient colitis as a result of on-target off-tumor destruction of CEA-expressing colonic mucosa (124). Ultimately, these studies demonstrated that while TCR T therapies targeting cancer differentiation antigens can mediate objective clinical responses, they are often associated with potentially dangerous on-target off-tumor toxicities. Likely owing to this, few TCR T trials targeting cancer differentiation antigens have been initiated in the past decade (**Supplementary Table 1**).

#### Trials Targeting NY-ESO-1: A Safe and Effective Target

The CGA NY-ESO-1 has been the most widely targeted antigen in TCR T trials, and several groups have now published promising results from trials targeting this antigen. An early landmark trial treating patients with refractory melanoma or synovial sarcoma with TCR T cells expressing an affinity-enhanced NY-ESO-1 specific TCR reported objective responses in 11/20 (55%) melanoma patients, including four durable complete responses, and 11/18 (61%) synovial sarcoma patients, including one durable complete response. No TCR T associated toxicities were observed (174). Later, a large study of synovial sarcoma patients treated with TCR T cells expressing an affinity enhanced NY-ESO-1 specific TCR (SPEAR T cells) observed clinical responses in 15/42 patients (36%), including one complete response, and 24/42 patients presenting with stable disease. These patients were also divided into treatment cohorts based on magnitude of NY-ESO-1 tumor expression and the lymphodepleting regimen they received. The greatest clinical responses were observed in a cohort of twelve patients (cohort 1) whose tumor expressed +2 or +3 NY-ESO-1 staining by immunohistochemistry in ≥50% of cells and who received a relatively intensive lymphodepleting regimen. Here, clinical responses were observed in 6/12 (50%) patients, including one complete response, and a median duration of response of 30.9 weeks (179). Of these twelve patients, five experienced cytokine release syndrome of grades 1 (2 pts), 2 (1 pt), or 3 (2 pts) (192). Several other studies reporting TCR T trials targeting NY-ESO-1 to treat various cancer types have also observed clinical responses without instances of on- or off- target toxicity attributed to TCR T cells (164, 176–178, 193). Together, these studies demonstrate that NY-ESO-1 targeting TCR T therapies are safe and capable of eliciting potent antitumor responses.

#### Trials Targeting MAGE-A Family Antigens: Early Toxicity and Recent Success

The MAGE-A family of proteins have also served as highly attractive CGA targets, and results from several TCR T trials targeting members of this family are now available. Unfortunately, two early reports of trials targeting MAGE-A3 described fatal TCR T mediated toxicity. In one study, two patients treated with TCR T cells expressing an affinity enhanced MAGE-A3 specific TCR died of cardiac toxicity following TCR T infusion. *Post hoc* investigation into the cause of these fatalities revealed cross-reactivity of the affinity enhanced TCR towards Titin expressed in cardiomyocytes (127, 128). This study emphasized the need for extensive preclinical investigation into potential off-target reactivities of lead TCRs. In a study published the same year, nine patients with metastatic cancer were treated with TCR T cells expressing an affinity enhanced TCR recognizing a similar MAGE-A3 and MAGE-A12 epitope. Clinical responses were achieved in 5/9 (56%) of patients, including a durable complete response in one patient. However, three patients experienced severe neurotoxicity following TCR T cell infusion, with two patients dying as a result. *Post hoc* analysis identified unexpected expression of MAGE-A12 in a subset of neurons in the brain, and the observed toxicity was thus presumed to be due to on-target off-tumor recognition of MAGE-A12 the brain (129). This study demonstrated the need for extensive preclinical characterization of cognate antigen targets in healthy tissue. A later study targeted MAGE-A3 with a high affinity HLA-DPB1*04:01 restricted TCR derived from a T regulatory cell. Objective responses were achieved in 4/17 (24%) of patients, with one patient achieving a durable complete response. Following TCR T treatment, one patient experienced grade 4 toxicities including increased ALT, AST, and creatinine, and eventually developed respiratory failure requiring hospitalization. A second patient experienced grade 3 toxicities of increased ALT, AST, and creatinine lasting two days. The cause of these toxicities was not described (194). Two recent reports of phase I trials of TCR T cells expressing an affinity-enhanced TCR specific for MAGE-A4 (ADP-A2M4 SPEAR T cells) to treat various solid cancers observed antitumor responses without evidence of serious TCR T mediated toxicity (182, 183). This year, Adaptimmune reported results of a phase II trial of ADP-A2M4 SPEAR T cells (now afamitresgene autoleucel) to treat patients with synovial sarcoma or myxoid/round cell liposarcoma (MRCLS). Here, objective responses were observed in 13/33 (39.4%) patients, including two durable complete responses, and disease control was achieved in 28/33 (84.8%) patients. Grades 1-2 CRS were observed in 21 patients and grade 3 CRS was observed in one patient. Based on this data, the company plans to file for afamitresgene autoleucel approval next year (184). In summary, results of early TCR T trials targeting MAGE-A3 demonstrated the need for more extensive preclinical testing of both on-target and off-target effects of lead TCRs and enhanced preclinical safety assessment strategies have emerged as a result of these studies. However, recent trials of Adaptimmune’s MAGE-A4-targeting afamitresgene autoleucel have demonstrated strong efficacy treating solid cancers in the absence of major TCR T cell mediated toxicity and may be nearing FDA approval.

#### Emerging Targets

Recent results have demonstrated efficacy of TCR T cells targeting HPV antigens in patients with various HPV-associated solid cancers. Relatively modest efficacy was achieved in a study treating twelve patients with TCR T cells expressing an E6-specific TCR, with objective responses observed in 2/12 (17%) patients, with no dose-limiting toxicities (186). In a related study using a higher affinity TCR recognizing E7, objective responses were achieved in 6/12 (50%) patients, with one dose-limiting toxicity that was presumably unrelated to TCR T mediated toxicity (44). However, the duration of clinical responses observed in both trials were relatively short (2 – 9 months). Recent studies have also demonstrated safety and efficacy of TCR T trials targeting the cancer overexpression antigen WT1. In one study, twelve AML patients at high risk for relapse following hematopoietic cell transplant (HCT) were treated with allogenic EBV-specific T cells engineered to express a WT1 specific TCR. Relapse free survival was achieved in 100% of the TCR T treated patients, as compared to 54% relapse free survival post-HCT in a comparative group of eighty-eight AML patients at similar risk of relapse. Nine patients exhibited grade 1-2 GVHD following TCR T infusion, with one patient developing grade 3 acute GVHD. However, the GVDH was determined to be most likely caused by the use of allogenic T cells rather than the introduced TCR (188).

#### Experiences of TCR T *vs* CAR T Trials to Treat Solid Tumors to Date

Recent years have seen the emergence of clinical trials reporting on the safety and efficacy of TCR T therapeutics in the context of both hematological malignancies and solid tumors (**Table 1**). Several TCR T trials focused on the latter have achieved improved therapeutic outcomes as compared to those achieved in CAR T trials (195), and it now appears that TCR T therapeutics are closer to receiving FDA approval for the treatment of solid cancers. A possible explanation for this disparity lies in the biological differences between TCRs and CARs (**Figure 1**), including 1) the ability of TCRs to recognize HLA-presented antigens derived from any cellular compartment including high specificity antigens such as CGAs and viral antigens, 2) TCRs are considerably more sensitive to low concentrations of a target antigen as compared to CARs, particularly in the case of affinity-enhanced TCRs (12–14), and 3) unlike CAR T cells, engineered TCRs do not drive ligand-independent tonic signaling (196, 197), making them better equipped to maintain function *in vivo*. Ultimately, the aforementioned factors are speculated to play a role in the comparatively improved performance of TCR T therapeutics targeting solid cancers, however, both fields are evolving rapidly with new antigen discoveries and further genetic engineering of the T cells, which should lead to improved efficacy in the upcoming solid tumor CAR T trials.

### Remaining Challenges

Intense efforts have been made to understand and address factors that limit the efficacy and clinical applicability of T cell-based ACTs, including TCR T therapy. This section describes select challenges currently facing TCR T therapy and the strategies available to meet them.

#### Manufacturing Cost and Complexity

TCR T products typically require 1-3 weeks of manufacture and involve several relatively complex processing steps that must be performed in highly regulated good manufacturing process (GMP) facilities. Despite the relative complexity, current TCR T manufacturing processes generally result in high rates of successful product manufacture. However, the cost of these T cell products is high. This is demonstrated by the high costs of FDA approved CAR T therapies, which utilize similar manufacturing processes. For example, single infusions of axicabtagene ciloleucel or tisagenlecleucel cost $373,000 and $475,000, respectively (198). In response to this, a strategy under development by many groups is the use allogeneic, or off-the-shelf, TCR T and CAR T products. However, a major challenge facing this approach is the likelihood of GVHD mediated by allogeneic T cells. Strategies to mediate GVHD of allogeneic T cell products include the use of oligoclonal virus-specific T cells with tightly restricted antigen specificities (188, 199), invariant T cell subsets such as γδ T cells (200) and iNKT cells (201), or *TRAC/TRBC* disrupted T cells (202). Another strategy to reduce the manufacturing cost of both TCR T and CAR T products is the use of non-viral gene delivery methods such as RNA electroporation (203, 204), transposons (205–207), and CRISPR/Cas9 (166).

#### T Cell Persistence

The ability of infused T cells to persist within the patient is an important factor mediating ACT antitumor efficacy (208). As such, intense efforts have been made to develop strategies that improve T cell persistence. Perhaps the most common strategy is to administer non-myeloablative lymphodepleting chemotherapy prior to T cell infusion, which alleviates competition of infused T cells with endogenous T cells for homeostatic cytokines such as IL-7 and IL-15, among other likely mechanisms (189). Several studies in mice have demonstrated that less differentiated T cell subsets (e.g., central memory and stem cell memory), have improved *in vivo* engraftment and persistence compared to highly differentiated T cells subsets (e.g., effector memory and terminally differentiated effectors) (209–211). As such, several strategies are now commonly employed to preserve less differentiated T cell subsets during ACT manufacture, including reduced expansion times and use of less differentiation-inducing cytokines (181, 212, 213). Some TCR T trials have further extended this approach by selecting specific T cell differentiation subsets prior to infusion (NCT02062359, NCT02408016). Another factor that limits T cell persistence is the paucity of costimulatory ligands within the tumor microenvironment. Several early TCR T trials performed coadministration of vaccines in efforts to provide TCR stimulation and costimulation *in vivo*. However, several of these studies failed to observe increased TCR T efficacy mediated by combination with vaccination (174, 176, 180). Genetic strategies to improve T cell costimulation include modified TCRs incorporating costimulatory domains (155) and coexpression of costim-only CARs (214) or domain swapped inhibitory receptors (215). Finally, several genetic strategies are also being explored to maintain T cell homeostatic cytokine signaling, including auto-secreting IL-15 or IL-12 elements (216–219) and a constitutively active IL-7 receptor (220).

#### Immunosuppressive Tumor Microenvironment

Another obstacle faced by adoptively transferred T cells is the immunosuppressive tumor microenvironment (TME). The TME supports tumor survival by recruiting immunosuppressive cell types, including myeloid derived suppressor cells (MDSCs), regulatory T cells (Tregs), and tumor-associated macrophages (TAMs) (221). Several pre-clinical studies of ACT are working to incorporate strategies to enable T cells to function in the hostile TME, including cotreatment with immune checkpoint blockade (222, 223), and genetic incorporation of inverted cytokine receptors (224) or MDSC targeting costimulatory receptors (214). A thorough understanding of the TME and its effect on T cells is necessary for the future success of ACT for solid cancers.

#### Tumor Intrinsic Escape Mechanisms

TCR T efficacy is influenced by the heterogeneity of cognate-antigen expression by tumor cells. This is especially the case when targeting CGAs, such as NY-ESO-1 and MAGE-A family proteins, which are often heterogeneously expressed by tumors (36). Nonetheless, complete responses have been observed in patients treated with TCR T cells targeting NY-ESO-1 and MAGE-A family proteins (**Table 1**). This may be partially explained by a phenomenon known as epitope spreading, whereby the immune response mounted by the infused TCR T cells leads to priming and activation of endogenous T cells to other non-cognate tumor antigens. Indeed, epitope spreading has been observed in humans following vaccination (225) and ACT (226, 227). Several genetic engineering strategies have now been developed to promote epitope spreading, including constitutively expressing inflammatory cytokines IL-12 (217, 218) and IL-18 (228), CD40L (229), or the DC growth factor FLT3 (230). Another approach to address antigen heterogeneity is to genetically encode specificity towards multiple antigens. While several multitargeting approaches have been developed in the CAR T realm (231), far less progress has been made in the development of multitargeting TCR T therapies to date.

TCR T therapies are also susceptible to tumor cell escape through perturbations in APM pathways. Downregulation of HLA-class I and other APM components such as TAP1 has been observed in many cancer types (232, 233). The impact of these escape mechanisms has been clearly demonstrated in TCR T and TIL trials where mutations in tumor APM components resulted in tumor escape and cancer progression (44, 67). These studies highlight an urgent need for strategies to address such tumor intrinsic mechanisms of escape, especially as we see further improvements of the antitumor efficacy of TCR T therapeutics. In cases of tumor downregulation of APM pathways, expression of these components may be recovered through administration of interferon (234) or epigenetic modifying drugs (235). Addressing cases where APM components are lost through hardwired genetic mutations is considerably more challenging. One strategy that has been investigated in mice is *in situ* gene delivery of *β2M* with an adenoviral vector (236). However, this approach delivered adenovirus into relatively small tumors (7 – 10 mm in diameter) by intratumoral injection, therefore it is unclear if this approach will be effective in patients whose tumors are large, inaccessible, and/or dispersed. Therefore, patients with hardwired loss of APM components will likely need to be treated with HLA-independent therapies such as CAR T. Interestingly, HLA-independent TCRs are also currently under development. These TCRs bind natively folded surface proteins similar to CARs, but possess binding affinities within the range of pMHC-TCR interactions (237).

## Conclusions

Immunotherapies have swiftly risen to become one of the major pillars in cancer treatment. Among them, TCR-engineered T cell therapies are a rapidly growing, active, and evolving field. Since the first report of redirected T cell specificity through TCR transfer in 1986 (238), tremendous progress has been made in TCR T therapies and their applications. Emerging technologies and enhanced strategies have made TCR discovery efforts considerably more time and cost effective. This will allow for more groups to become involved in TCR discovery and will ultimately lead to an increase in TCRs targeting new tumor antigens and restricted to a broader range of HLAs. Several clinical findings from early TCR T trials have shaped the past decade of TCR T development. Toxicities observed in early trials have led to improved preclinical safety assessments of TCRs and a transition towards antigen targets with increased tumor specificity. Since 2015 there has been an influx of results of TCR T trials treating various solid cancers and hematological malignancies. Several of these trials have demonstrated impressive clinical responses in the absence of serious toxicities, and it now seems that the first approval of TCR T therapies for solid cancer may be around the corner. These early results give reason for optimism in the continued development of TCR T therapies for cancer.

## Author Contributions

PS and LK contributed to the development, writing, and review of the article. VH contributed to the figures and review of the article. All authors contributed to the article and approved the submitted version.

## Funding

Cancer Prevention and Research Institute of Texas (CPRIT) Grant ID: RR170024 and the Susan Komen Career Catalyst Research grant # CCR19606699.

## Conflict of Interest

PS and VH have submitted a patent application for TCR discovery.

The remaining author declares that the research was conducted in the absence of any commercial or financial relationships that could be construed as a potential conflict of interest.

## Publisher’s Note

All claims expressed in this article are solely those of the authors and do not necessarily represent those of their affiliated organizations, or those of the publisher, the editors and the reviewers. Any product that may be evaluated in this article, or claim that may be made by its manufacturer, is not guaranteed or endorsed by the publisher.

## References

[1] Rosenberg SA Packard BS Aebersold PM Solomon D Topalian SL Toy ST. Use of Tumor-Infiltrating Lymphocytes and Interleukin-2 in the Immunotherapy of Patients With Metastatic Melanoma. A Preliminary Report. N Engl J Med (1988) 319:1676–80. doi: 10.1056/NEJM198812223192527 3264384

[2] Weber EW Maus MV Mackall CL. The Emerging Landscape of Immune Cell Therapies. Cell (2020) 181:46–62. doi: 10.1016/j.cell.2020.03.001 32243795 PMC8900215

[3] Garber K. Driving T-Cell Immunotherapy to Solid Tumors. Nat Biotechnol (2018) 36:215–9. doi: 10.1038/nbt.4090 29509745

[4] Rudolph MG Stanfield RL Wilson IA. How TCRs Bind MHCs, Peptides, and Coreceptors. Annu Rev Immunol (2006) 24:419–66. doi: 10.1146/annurev.immunol.23.021704.115658 16551255

[5] Huang Y Wange RL. T Cell Receptor Signaling: Beyond Complex Complexes. J Biol Chem (2004) 279:28827–30. doi: 10.1074/JBC.R400012200 15084594

[6] Anton van der Merwe P Dushek O. Mechanisms for T Cell Receptor Triggering. Nat Rev Immunol (2011) 11:47–55. doi: 10.1038/nri2887 21127503

[7] Davis MM. T Cell Receptor Gene Diversity and Selection. Annu Rev Biochem (1990) 59:475–96. doi: 10.1146/annurev.bi.59.070190.002355 2197981

[8] Mørch AM Bálint Š Santos AM Davis SJ Dustin ML. Coreceptors and TCR Signaling – the Strong and the Weak of it. Front Cell Dev Biol (2020) 8:597627. doi: 10.3389/fcell.2020.597627 33178706 PMC7596257

[9] Abate-Daga D Davila ML. CAR Models: Next-Generation CAR Modifications for Enhanced T-Cell Function. Mol Ther - Oncolytics (2016) 3:16014. doi: 10.1038/MTO.2016.14 27231717 PMC4871190

[10] Robinson J Halliwell JA Hayhurst JD Flicek P Parham P Marsh SGE. The IPD and IMGT/HLA Database: Allele Variant Databases. Nucleic Acids Res (2015) 43:D423–31. doi: 10.1093/NAR/GKU1161 PMC4383959 25414341

[11] Gonzalez-Galarza FF McCabe A Melo dos Santos EJ Jones J Takeshita L Ortega-Rivera ND. Allele Frequency Net Database (AFND) 2020 Update: Gold-Standard Data Classification, Open Access Genotype Data and New Query Tools. Nucleic Acids Res (2020) 48:D783–8. doi: 10.1093/NAR/GKZ1029 PMC7145554 31722398

[12] Chandran SS Klebanoff CA. T Cell Receptor-Based Cancer Immunotherapy: Emerging Efficacy and Pathways of Resistance. Immunol Rev (2019) 290:127–47. doi: 10.1111/IMR.12772 PMC7027847 31355495

[13] Salter AI Rajan A Kennedy JJ Ivey RG Shelby SA Leung I. Comparative Analysis of TCR and CAR Signaling Informs CAR Designs With Superior Antigen Sensitivity and In Vivo Function. Sci Signal (2021) 14:eabe2606. doi: 10.1126/scisignal.abe2606 34429382 PMC8613804

[14] Harris DT Hager MV Smith SN Cai Q Stone JD Kruger P. Comparison of T Cell Activities Mediated by Human TCRs and CARs That Use the Same Recognition Domains. J Immunol (2018) 200:1088–100. doi: 10.4049/JIMMUNOL.1700236 PMC5780198 29288199

[15] Majzner RG Mackall CL. Tumor Antigen Escape From CAR T-Cell Therapy. Cancer Discovery (2018) 8:1219–26. doi: 10.1158/2159-8290.CD-18-0442 30135176

[16] Rapoport AP Stadtmauer EA Binder-Scholl GK Goloubeva O Vogl DT Lacey SF. NY-ESO-1–Specific TCR–engineered T Cells Mediate Sustained Antigen-Specific Antitumor Effects in Myeloma. Nat Med (2015) 21:914–21. doi: 10.1038/nm.3910 PMC4529359 26193344

[17] Frey N Porter D. Cytokine Release Syndrome With Chimeric Antigen Receptor T Cell Therapy. Biol Blood Marrow Transplant (2019) 25:e123–7. doi: 10.1016/J.BBMT.2018.12.756 30586620

[18] Fagerberg L Jonasson K Von Heijne G Uhlén M Berglund L. Prediction of the Human Membrane Proteome. Proteomics (2010) 10:1141–9. doi: 10.1002/PMIC.200900258 20175080

[19] Uhlén M Fagerberg L Hallström BM Lindskog C Oksvold P Mardinoglu A. Tissue-Based Map of the Human Proteome. Science (2015) 347:1260419. doi: 10.1126/science.1260419 25613900

[20] Bausch-Fluck D Goldmann U Müller S van Oostrum M Müller M Schubert OT. The in Silico Human Surfaceome. Proc Natl Acad Sci USA (2018) 115:E10988–97. doi: 10.1073/pnas.1808790115 PMC6243280 30373828

[21] Ponterio E De Maria R Haas TL. Identification of Targets to Redirect CAR T Cells in Glioblastoma and Colorectal Cancer: An Arduous Venture. Front Immunol (2020) 11:565631. doi: 10.3389/fimmu.2020.565631 33101285 PMC7555836

[22] Lim WA June CH. The Principles of Engineering Immune Cells to Treat Cancer. Cell (2017) 168:724–40. doi: 10.1016/j.cell.2017.01.016 PMC5553442 28187291

[23] Watanabe K Kuramitsu S Posey ADJ June CH. Expanding the Therapeutic Window for CAR T Cell Therapy in Solid Tumors: The Knowns and Unknowns of CAR T Cell Biology. Front Immunol (2018) 9:2486. doi: 10.3389/FIMMU.2018.02486 30416506 PMC6212550

[24] Vigneron N. Human Tumor Antigens and Cancer Immunotherapy. BioMed Res Int (2015) 2015:948501. doi: 10.1155/2015/948501 26161423 PMC4487697

[25] Ilyas S Yang JC. Landscape of Tumor Antigens in T Cell Immunotherapy. J Immunol (2015) 195:5117–22. doi: 10.4049/jimmunol.1501657 PMC4656134 26589749

[26] Coulie PG Van den Eynde BJ van der Bruggen P Boon T. Tumour Antigens Recognized by T Lymphocytes: At the Core of Cancer Immunotherapy. Nat Rev Cancer (2014) 14:135–46. doi: 10.1038/nrc3670 24457417

[27] Kawakami Y Eliyahu S Delgado CH Robbins PF Rivoltini L Topalian SL. Cloning of the Gene Coding for a Shared Human Melanoma Antigen Recognized by Autologous T Cells Infiltrating Into Tumor. Proc Natl Acad Sci USA (1994) 91:3515–9. doi: 10.1073/pnas.91.9.3515 PMC43610 8170938

[28] Bakker ABH Schreurs MWJ De Boer AJ Kawakami Y Rosenberg SA Adema GJ. Melanocyte Lineage-Specific Antigen Gp100 Is Recognized by Melanoma-Derived Tumor-Infiltrating Lymphocytes. J Exp Med (1994) 179:1005–9. doi: 10.1084/jem.179.3.1005 PMC2191413 8113668

[29] Cilloni D Gottardi E De Micheli D Serra A Volpe G Messa F. Quantitative Assessment of WT1 Expression by Real Time Quantitative PCR may be a Useful Tool for Monitoring Minimal Residual Disease in Acute Leukemia Patients. Leukemia (2002) 16:2115–21. doi: 10.1038/sj.leu.2402675 12357365

[30] Inoue K Ogawa H Sonoda Y Kimura T Sakabe H Oka Y. Aberrant Overexpression of the Wilms Tumor Gene (WT1) in Human Leukemia. Blood (1997) 89:1405–12. doi: 10.1182/BLOOD.V89.4.1405 9028964

[31] Chen Y-T Scanlan MJ Sahin U Tureci O Gure AO Tsang S. A Testicular Antigen Aberrantly Expressed in Human Cancers Detected by Autologous Antibody Screening. Proc Natl Acad Sci U.S.A. (1997) 94:1914–8. doi: 10.1073/pnas.94.5.1914 PMC20017 9050879

[32] Dhodapkar MV Osman K Teruya-Feldstein J Filippa D Hedvat CV Iversen K. Expression of Cancer/Testis (CT) Antigens MAGE-A1, MAGE-A3, MAGE-A4, CT-7, and NY-ESO-1 in Malignant Gammopathies is Heterogeneous and Correlates With Site, Stage and Risk Status of Disease. Cancer Immun Arch (2003) 3:9. 12875607

[33] Bergeron A Picard V LaRue H Harel F Hovington H Lacombe L. High Frequency of MAGE-A4 and MAGE-A9 Expression in High-Risk Bladder Cancer. Int J Cancer (2009) 125:1365–71. doi: 10.1002/IJC.24503 19533752

[34] Duffour M-T Chaux P Lurquin C Cornelis G Boon T van der Bruggen P. A MAGE-A4 Peptide Presented by HLA-A2 is Recognized by Cytolytic T Lymphocytes. Eur J Immunol (1999) 29:3329–37. doi: 10.1002/(SICI)1521-4141(199910)29:10<3329::AID-IMMU3329>3.0.CO;2-7 10540345

[35] Yakirevich E Sabo E Lavie O Mazareb S Spagnoli GC Resnick MB. Expression of the MAGE-A4 and NY-ESO-1 Cancer-Testis Antigens in Serous Ovarian Neoplasms. Clin Cancer Res (2003) 9:6453–60. 14695148

[36] Scanlan MJ Gure AO Jungbluth AA Old LJ Chen Y-T. Cancer/testis Antigens: An Expanding Family of Targets for Cancer. Immunol Rev (2002) 188:22–32. doi: 10.1034/j.1600-065x.2002.18803.x 12445278

[37] de Martel C Plummer M Vignat J Franceschi S. Worldwide Burden of Cancer Attributable to HPV by Site, Country and HPV Type. Int J Cancer (2017) 141:664–70. doi: 10.1002/IJC.30716 PMC5520228 28369882

[38] Perz JF Armstrong GL Farrington LA Hutin YJF Bell BP. The Contributions of Hepatitis B Virus and Hepatitis C Virus Infections to Cirrhosis and Primary Liver Cancer Worldwide. J Hepatol (2006) 45:529–38. doi: 10.1016/J.JHEP.2006.05.013 16879891

[39] Thompson MP Kurzrock R. Epstein-Barr Virus and Cancer. Clin Cancer Res (2004) 10:803–21. doi: 10.1158/1078-0432.CCR-0670-3 14871955

[40] Khan G Hashim MJ. Global Burden of Deaths From Epstein-Barr Virus Attributable Malignancies 1990-2010. Infect Agent Cancer (2014) 9:38. doi: 10.1186/1750-9378-9-38 25473414 PMC4253616

[41] Pal A Kundu R. Human Papillomavirus E6 and E7: The Cervical Cancer Hallmarks and Targets for Therapy. Front Microbiol (2020) 10:3116. doi: 10.3389/FMICB.2019.03116 32038557 PMC6985034

[42] Yim E-K Park J-S. The Role of HPV E6 and E7 Oncoproteins in HPV-Associated Cervical Carcinogenesis. Cancer Res Treat (2005) 37:319–24. doi: 10.4143/CRT.2005.37.6.319 PMC2785934 19956366

[43] Dawson CW Port RJ Young LS. The Role of the EBV-Encoded Latent Membrane Proteins LMP1 and LMP2 in the Pathogenesis of Nasopharyngeal Carcinoma (NPC). Semin Cancer Biol (2012) 22:144–53. doi: 10.1016/J.SEMCANCER.2012.01.004 22249143

[44] Nagarsheth NB Norberg SM Sinkoe AL Adhikary S Meyer TJ Lack JB. TCR-Engineered T Cells Targeting E7 for Patients With Metastatic HPV-Associated Epithelial Cancers. Nat Med (2021) 27:419–25. doi: 10.1038/s41591-020-01225-1 PMC9620481 33558725

[45] Draper LM Kwong ML Gros A Stevanović S Tran E Kerkar S. Targeting of HPV-16+ Epithelial Cancer Cells by TCR Gene Engineered T Cells Directed Against E6. Clin Cancer Res (2015) 21:4431–9. doi: 10.1158/1078-0432.CCR-14-3341 PMC4603283 26429982

[46] Cho H-I Kim U-H Shin A-R Won J-N Lee H-J Sohn H-J. A Novel Epstein–Barr Virus-Latent Membrane Protein-1-Specific T-Cell Receptor for TCR Gene Therapy. Br J Cancer (2018) 118:534–45. doi: 10.1038/BJC.2017.475 PMC5830600 29360818

[47] Gottschalk S Heslop H Rooney C. Adoptive Immunotherapy for EBV-Associated Malignancies. Leuk Lymphoma (2005) 46:1–10. doi: 10.1080/10428190400002202 15621775

[48] Bollard CM Gottschalk S Torrano V Diouf O Ku S Hazrat Y. Sustained Complete Responses in Patients With Lymphoma Receiving Autologous Cytotoxic T Lymphocytes Targeting Epstein-Barr Virus Latent Membrane Proteins. J Clin Oncol (2014) 32:798–808. doi: 10.1200/JCO.2013.51.5304 24344220 PMC3940538

[49] Cho SG Kim N Sohn HJ Lee SK Oh ST Lee HJ. Long-Term Outcome of Extranodal Nk/T Cell Lymphoma Patients Treated With Postremission Therapy Using EBV LMP1 and LMP2a-Specific CTLs. Mol Ther (2015) 23:1401–9. doi: 10.1038/MT.2015.91 PMC4817864 26017177

[50] Negrini S Gorgoulis VG Halazonetis TD. Genomic Instability — an Evolving Hallmark of Cancer. Nat Rev Mol Cell Biol (2010) 11:220–8. doi: 10.1038/nrm2858 20177397

[51] Bailey MH Tokheim C Porta-Pardo E Sengupta S Bertrand D Weerasinghe A. Comprehensive Characterization of Cancer Driver Genes and Mutations. Cell (2018) 173:371–85. doi: 10.1016/j.cell.2018.02.060 PMC6029450 29625053

[52] Stratton MR Campbell PJ Futreal PA. The Cancer Genome. Nature (2009) 458:719–24. doi: 10.1038/NATURE07943 PMC2821689 19360079

[53] Martínez-Jiménez F Muiños F Sentís I Deu-Pons J Reyes-Salazar I Arnedo-Pac C. A Compendium of Mutational Cancer Driver Genes. Nat Rev Cancer (2020) 20:555–72. doi: 10.1038/s41568-020-0290-x 32778778

[54] Reiter JG Makohon-Moore AP Gerold JM Heyde A Attiyeh MA Kohutek ZA. Minimal Functional Driver Gene Heterogeneity Among Untreated Metastases. Science (2018) 361:1033–7. doi: 10.1126/SCIENCE.AAT7171 PMC6329287 30190408

[55] Armistead PM. Cellular Therapy Against Public Neoantigens. J Clin Invest (2019) 129:506–8. doi: 10.1172/JCI126116 PMC6355209 30640175

[56] Pearlman AH Hwang MS Konig MF Hsiue EH-C Douglass J DiNapoli SR. Targeting Public Neoantigens for Cancer Immunotherapy. Nat Cancer (2021) 2:487–97. doi: 10.1038/s43018-021-00210-y PMC8525885 34676374

[57] Klebanoff CA Wolchok JD. Shared Cancer Neoantigens: Making Private Matters Public. J Exp Med (2018) 215:5–7. doi: 10.1084/JEM.20172188 29269561 PMC5748868

[58] Wang QJ Yu Z Griffith K Hanada K Restifo NP Yang JC. Identification of T-Cell Receptors Targeting KRAS-Mutated Human Tumors. Cancer Immunol Res (2016) 4:204–14. doi: 10.1158/2326-6066.CIR-15-0188 PMC4775432 26701267

[59] Spoerke JM Gendreau S Walter K Qiu J Wilson TR Savage H. Heterogeneity and Clinical Significance of ESR1 Mutations in ER-Positive Metastatic Breast Cancer Patients Receiving Fulvestrant. Nat Commun (2016) 7:1–10. doi: 10.1038/ncomms11579 PMC4869259 27174596

[60] Chandran SS Ma J Klatt MG Dundar F Bandlamudi C Razavi P. Immunogenicity of a Public Neoantigen Derived From Mutated PIK3CA. bioRxiv (2021). doi: 10.1101/2021.04.08.439061 PMC9117146 35484264

[61] Lythe G Callard RE Hoare RL Molina-París C. How Many TCR Clonotypes Does a Body Maintain? J Theor Biol (2016) 389:214–24. doi: 10.1016/j.jtbi.2015.10.016 PMC4678146 26546971

[62] Balch CM Riley LB Bae YJ Salmeron MA Platsoucas CD von EA. Patterns of Human Tumor-Infiltrating Lymphocytes in 120 Human Cancers. Arch Surg (1990) 125:200–5. doi: 10.1001/ARCHSURG.1990.01410140078012 1689143

[63] Jin BY Campbell TE Draper LM Stevanović S Weissbrich B Yu Z. Engineered T Cells Targeting E7 Mediate Regression of Human Papillomavirus Cancers in a Murine Model. JCI Insight (2018) 3:e99499. doi: 10.1172/jci.insight.99488 PMC5931134 29669936

[64] Lu YC Zheng Z Robbins PF Tran E Prickett TD Gartner JJ. An Efficient Single-Cell RNA-Seq Approach to Identify Neoantigen-Specific T Cell Receptors. Mol Ther (2018) 26:379–89. doi: 10.1016/J.YMTHE.2017.10.018 PMC5835023 29174843

[65] Lu Y-C Zheng Z Lowery FJ Gartner JJ Prickett TD Robbins PF. Direct Identification of Neoantigen-Specific TCRs From Tumor Specimens by High-Throughput Single-Cell Sequencing. J Immunother Cancer (2021) 9:e002595. doi: 10.1136/jitc-2021-002595 34321276 PMC8320258

[66] Zacharakis N Chinnasamy H Black M Xu H Lu YC Zheng Z. Immune Recognition of Somatic Mutations Leading to Complete Durable Regression in Metastatic Breast Cancer. Nat Med (2018) 24:724–30. doi: 10.1038/s41591-018-0040-8 PMC6348479 29867227

[67] Tran E Robbins PF Lu YC Prickett TD Gartner JJ Jia L. T-Cell Transfer Therapy Targeting Mutant KRAS in Cancer. N Engl J Med (2016) 375:2255–62. doi: 10.1056/NEJMoa1609279 PMC5178827 27959684

[68] Rosenberg SA Yannelli JR Yang JC Topalian SL Schwartzentruber DJ Weber JS. Treatment of Patients With Metastatic Melanoma With Autologous Tumor-Infiltrating Lymphocytes and Interleukin 2. JNCI J Natl Cancer Inst (1994) 86:1159–66. doi: 10.1093/JNCI/86.15.1159 8028037

[69] Rosenberg SA Restifo NP Yang JC Morgan RA Dudley ME. Adoptive Cell Transfer: A Clinical Path to Effective Cancer Immunotherapy. Nat Rev Cancer (2008) 8:299–308. doi: 10.1038/NRC2355 18354418 PMC2553205

[70] Wang S Sun J Chen K Ma P Lei Q Xing S. Perspectives of Tumor-Infiltrating Lymphocyte Treatment in Solid Tumors. BMC Med (2021) 19:1–7. doi: 10.1186/S12916-021-02006-4 34112147 PMC8194199

[71] Lee S Psyrri A Sukari A Thomas S Wenham R Gogas H. Phase 2 Efficacy and Safety of Autologous Tumor-Infiltrating Lymphocyte (TIL) Cell Therapy in Combination With Pembrolizumab in Immune Checkpoint Inhibitor-Naïve Patients With Advanced Cancers [Conference Presentation]. In: SITC. Washington, D.C., United States: J ImmunoTher Cancer (2021). doi: 10.1136/jitc-2021-SITC2021.492

[72] Chinnasamy N Wargo JA Yu Z Rao M Frankel TL Riley JP. A TCR Targeting the HLA-A*0201–Restricted Epitope of MAGE-A3 Recognizes Multiple Epitopes of the MAGE-A Antigen Superfamily in Several Types of Cancer. J Immunol (2011) 186:685–96. doi: 10.4049/JIMMUNOL.1001775 PMC6292200 21149604

[73] Kunert A van Brakel M van Steenbergen-Langeveld S da Silva M Coulie PG Lamers C. MAGE-C2–Specific TCRs Combined With Epigenetic Drug-Enhanced Antigenicity Yield Robust and Tumor-Selective T Cell Responses. J Immunol (2016) 197:2541–52. doi: 10.4049/JIMMUNOL.1502024 27489285

[74] Abrams SI Khleif SN Bergmann-Leitner ES Kantor JA Chung Y Hamilton JM. Generation of Stable CD4+ and CD8+ T Cell Lines From Patients Immunized With Ras Oncogene-Derived Peptides Reflecting Codon 12 Mutations. Cell Immunol (1997) 182:137–51. doi: 10.1006/CIMM.1997.1224 9514698

[75] Heslop PIHE Brenner MK Rooney C Krance C-IRA Roberts WM Rochester R. Administration of Neomycin Resistance Gene Marked EBV Specific Cytotoxic T Lymphocytes to Recipients of Mismatched-Related or Phenotypically Similar Unrelated Donor Marrow Grafts. Hum Gene Ther (1994) 5:381–97. doi: 10.1089/HUM.1994.5.3-381 8018749

[76] Greenberg SR Riddell M Rabin AP Geballe WJ Britt PD. Class I MHC-Restricted Cytotoxic T Lymphocyte Recognition of Cells Infected With Human Cytomegalovirus Does Not Require Endogenous Viral Gene Expression(1991). Available at: http://www.jimmunol.org/content/146/8/2795 (Accessed October 19, 2021). 1707922

[77] Reusser P Riddell S Meyers J Greenberg P. Cytotoxic T-Lymphocyte Response to Cytomegalovirus After Human Allogeneic Bone Marrow Transplantation: Pattern of Recovery and Correlation With Cytomegalovirus Infection and Disease. Blood (1991) 78:1373–80. doi: 10.1182/BLOOD.V78.5.1373.1373 1652311

[78] Laubscher A Bluestein HG Spector SA Zvaifler NJ. Generation of Human Cytomegalovirus-Specific Cytotoxic T-Lymphocytes in a Short-Term Culture. J Immunol Methods (1988) 110:69–77. doi: 10.1016/0022-1759(88)90084-1 2836515

[79] Bethune MT Li X-H Yu J McLaughlin J Cheng D Mathis C. Isolation and Characterization of NY-ESO-1-Specific T Cell Receptors Restricted on Various MHC Molecules. Proc Natl Acad Sci USA (2018) 115:E10702–11. doi: 10.1073/pnas.1810653115 PMC6233129 30348802

[80] Gerdemann U Katari U Christin AS Cruz CR Tripic T Rousseau A. Cytotoxic T Lymphocytes Simultaneously Targeting Multiple Tumor-Associated Antigens to Treat EBV Negative Lymphoma. Mol Ther (2011) 19:2258–68. doi: 10.1038/mt.2011.167 PMC3242656 21915103

[81] Leung WK Workineh A Mukhi S Tzannou I Brenner D Watanabe N. Evaluation of Cyclin A1-Specific T Cells as a Potential Treatment for Acute Myeloid Leukemia. Blood Adv (2020) 4:387–97. doi: 10.1182/bloodadvances.2019000715 PMC6988407 31985805

[82] Cafri G Yossef R Pasetto A Deniger DC Lu YC Parkhurst M. Memory T Cells Targeting Oncogenic Mutations Detected in Peripheral Blood of Epithelial Cancer Patients. Nat Commun (2019) 10:1–9. doi: 10.1038/s41467-019-08304-z 30683863 PMC6347629

[83] Malekzadeh P Yossef R Cafri G Paria BC Lowery FJ Jafferji M. Antigen Experienced T Cells From Peripheral Blood Recognize P53 Neoantigens. Clin Cancer Res (2020) 26:1267–76. doi: 10.1158/1078-0432.ccr-19-1874 PMC7424598 31996390

[84] Arber C Feng X Abhyankar H Romero E Wu M-F Heslop HH. Survivin-Specific T Cell Receptor Targets Tumor But Not T Cells. J Clin Invest (2015) 125:157–68. doi: 10.1172/JCI75876 PMC4382259 25415440

[85] Inozume T Hanada K Wang QJ Ahmadzadeh M Wunderlich JR Rosenberg SA. Selection of CD8+PD-1+ Lymphocytes in Fresh Human Melanomas Enriches for Tumor-Reactive T-Cells. J Immunother (2010) 33:956–64. doi: 10.1097/CJI.0B013E3181FAD2B0 PMC2980947 20948441

[86] Gros A Parkhurst MR Tran E Pasetto A Robbins PF Ilyas S. Prospective Identification of Neoantigen-Specific Lymphocytes in the Peripheral Blood of Melanoma Patients. Nat Med (2016) 22:433–8. doi: 10.1038/NM.4051 PMC7446107 26901407

[87] Gros A Robbins PF Yao X Li YF Turcotte S Tran E. PD-1 Identifies the Patient-Specific CD8^+^ Tumor-Reactive Repertoire Infiltrating Human Tumors. J Clin Invest (2014) 124:2246–59. doi: 10.1172/JCI73639 PMC4001555 24667641

[88] Butler MO Hirano N. Human Cell-Based Artificial Antigen-Presenting Cells for Cancer Immunotherapy. Immunol Rev (2014) 257:191–209. doi: 10.1111/IMR.12129 24329798 PMC3869003

[89] Kim JV Latouche J-B Rivière I Sadelain M. The ABCs of Artificial Antigen Presentation. Nat Biotechnol (2004) 22:403–10. doi: 10.1038/nbt955 15060556

[90] Neal LR Bailey SR Wyatt MM Bowers JS Majchrzak K Nelson MH. The Basics of Artificial Antigen Presenting Cells in T Cell-Based Cancer Immunotherapies. J Immunol Res Ther (2017) 2:68–79. 28825053 PMC5560309

[91] Sprouse ML Blahnik G Lee T Tully N Benarjee P James EA. Streamlined Single Cell TCR Isolation and Generation of Retroviral Vectors for *In Vitro* and *In Vivo* Expression of Human TCRs. JoVE (J Vis Exp (2017) 127:e55379. doi: 10.3791/55379 PMC5752194 28930975

[92] Rosati SF Parkhurst MR Hong Y Zheng Z Feldman SA Rao M. A Novel Murine T-Cell Receptor Targeting NY-ESO-1. J Immunother (2014) 37:135–46. doi: 10.1097/CJI.0000000000000019 PMC7443746 24598449

[93] Van Der Bruggen P Russo E Schultz GR Cornelis T Boon V Stroobant V. Identification of Five MAGE-A1 Epitopes Recognized by Cytolytic T Lymphocytes Obtained by *In Vitro* Stimulation With Dendritic Cells Transduced With MAGE-A1(1999). Available at: http://www.jimmunol.org/content/163/5/2928.full (Accessed October 17, 2021). 10453041

[94] Mariotti S Nisini R. Generation of Human T Cell Clones. TC ell Protoc Second Ed (2009) 514:65–93. doi: 10.1007/978-1-60327-527-9_6 19048214

[95] Verhoef A. Production of Human T-Cell Clones. Methods Mol Med Allergy Methods Protoc (2008) 138:43–50. doi: 10.1007/978-1-59745-366-0_4 18615242

[96] Thompson T Boon PD Greenberg C Yee MJ Gilbert SR Riddell VG. Isolation of Tyrosinase-Specific CD8+ and CD4+ T Cell Clones From the Peripheral Blood of Melanoma Patients Following In Vitro Stimulation With Recombinant Vaccinia Virus(1996). Available at: http://www.jimmunol.org/content/157/9/4079 (Accessed November 4, 2020). 8892642

[97] Ho WY Nguyen HN Wolfl M Kuball J Greenberg PD. *In Vitro* Methods for Generating CD8+ T-Cell Clones for Immunotherapy From the Naïve Repertoire. J Immunol Methods (2006) 310:40–52. doi: 10.1016/j.jim.2005.11.023 16469329

[98] Pasetto A Gros A Robbins PF Deniger DC Prickett TD Matus-Nicodemos R. Tumor- and Neoantigen-Reactive T-Cell Receptors Can Be Identified Based on Their Frequency in Fresh Tumor. Cancer Immunol Res (2016) 4:734–43. doi: 10.1158/2326-6066.CIR-16-0001 PMC5010958 27354337

[99] Lulla PD Tzannou I Vasileiou S Carrum G Ramos CA Kamble R. The Safety and Clinical Effects of Administering a Multiantigen-Targeted T Cell Therapy to Patients With Multiple Myeloma. Sci Transl Med (2020) 12:eaaz3339. doi: 10.1126/SCITRANSLMED.AAZ3339 32727914

[100] Parkhurst M Gros A Pasetto A Prickett T Crystal JS Robbins P. Isolation of T-Cell Receptors Specifically Reactive With Mutated Tumor-Associated Antigens From Tumor-Infiltrating Lymphocytes Based on CD137 Expression. Clin Cancer Res (2017) 23:2491–505. doi: 10.1158/1078-0432.CCR-16-2680 PMC6453117 27827318

[101] Ma W Vigneron N Chapiro J Stroobant V Germeau C Boon T. A MAGE-C2 Antigenic Peptide Processed by the Immunoproteasome is Recognized by Cytolytic T Cells Isolated From a Melanoma Patient After Successful Immunotherapy. Int J Cancer (2011) 129:2427–34. doi: 10.1002/IJC.25911 21207413

[102] Kawakami Y Eliyahu S Delgado CH Robbins PF Sakaguchi K Appella E. Identification of a Human Melanoma Antigen Recognized by Tumor-Infiltrating Lymphocytes Associated With *In Vivo* Tumor Rejection. Proc Natl Acad Sci USA (1994) 91:6458. doi: 10.1073/PNAS.91.14.6458 8022805 PMC44221

[103] Tran E Ahmadzadeh M Lu Y-C Gros A Turcotte S Robbins PF. Immunogenicity of Somatic Mutations in Human Gastrointestinal Cancers. Science (2015) 350:1387–90. doi: 10.1126/science.aad1253 PMC7445892 26516200

[104] Lu YC Yao X Crystal JS Li YF El-Gamil M Gross C. Efficient Identification of Mutated Cancer Antigens Recognized by T Cells Associated With Durable Tumor Regressions. Clin Cancer Res (2014) 20:3401–10. doi: 10.1158/1078-0432.CCR-14-0433 PMC4083471 24987109

[105] Hebeisen M Allard M Gannon PO Schmidt J Speiser DE Rufer N. Identifying Individual T Cell Receptors of Optimal Avidity for Tumor Antigens. Front Immunol (2015) 6:582. doi: 10.3389/FIMMU.2015.00582 26635796 PMC4649060

[106] Spear TT Evavold BD Baker BM Nishimura MI. Understanding TCR Affinity, Antigen Specificity, and Cross-Reactivity to Improve TCR Gene-Modified T Cells for Cancer Immunotherapy. Cancer Immunol Immunother (2019) 68:1881–9. doi: 10.1007/s00262-019-02401-0 PMC11028285 31595324

[107] Stone JD Chervin AS Kranz DM. T-Cell Receptor Binding Affinities and Kinetics: Impact on T-Cell Activity and Specificity. Immunology (2009) 126:165. doi: 10.1111/J.1365-2567.2008.03015.X 19125887 PMC2632691

[108] Schmid DA Irving MB Posevitz V Hebeisen M Posevitz-Fejfar A Sarria J-CF. Evidence for a TCR Affinity Threshold Delimiting Maximal CD8 T Cell Function. J Immunol (2010) 184:4936–46. doi: 10.4049/jimmunol.1000173 20351194

[109] Zhao Y Bennett AD Zheng Z Wang QJ Robbins PF Yu LYL. High-Affinity TCRs Generated by Phage Display Provide CD4 + T Cells With the Ability to Recognize and Kill Tumor Cell Lines. J Immunol (2007) 179:5845–54. doi: 10.4049/jimmunol.179.9.5845 PMC2140228 17947658

[110] Zhong S Malecek K Johnson LA Yu Z de Miera EV-S Darvishian F. T-Cell Receptor Affinity and Avidity Defines Antitumor Response and Autoimmunity in T-Cell Immunotherapy. Proc Natl Acad Sci (2013) 110:6973–8. doi: 10.1073/PNAS.1221609110 PMC3637771 23576742

[111] Hu Z Zhu L Wang J Wan Y Yuan S Chen J. Immune Signature of Enhanced Functional Avidity CD8+ T Cells *In Vivo* Induced by Vaccinia Vectored Vaccine. Sci Rep (2017) 7:1–12. doi: 10.1038/srep41558 28155878 PMC5290741

[112] Jurtz V Paul S Andreatta M Marcatili P Peters B Nielson M. NetMHCpan-4.0: Improved Peptide-MHC Class I Interaction Predictions Integrating Eluted Ligand and Peptide Binding Affinity Data. J Immunol (2017) 199:3360–8. doi: 10.4049/JIMMUNOL.1700893 PMC5679736 28978689

[113] Marcu A Bichmann L Kuchenbecker L Kowalewski DJ Freudenmann LK Backert L. HLA Ligand Atlas: A Benign Reference of HLA-Presented Peptides to Improve T-Cell-Based Cancer Immunotherapy. J Immunother Cancer (2021) 9:e002071. doi: 10.1136/JITC-2020-002071 33858848 PMC8054196

[114] Tanaka K. The Proteasome: Overview of Structure and Functions. Proc Jpn Acad (2009) 85:12–36. doi: 10.2183/PJAB.85.12 PMC3524306 19145068

[115] Guillaume B Chapiro J Stroobant V Colau D Van HB Parvizi G. Two Abundant Proteasome Subtypes That Uniquely Process Some Antigens Presented by HLA Class I Molecules. Proc Natl Acad Sci (2010) 107:18599–604. doi: 10.1073/PNAS.1009778107 PMC2972972 20937868

[116] Schultz E Chapiro J Lurquin C Claverol S Burlet-Schiltz O Warnier G. The Production of a New MAGE-3 Peptide Presented to Cytolytic T Lymphocytes by HLA-B40 Requires the Immunoproteasome. J Exp Med (2002) 195:391–9. doi: 10.1084/JEM.20011974 PMC2193621 11854353

[117] Guillaume B Stroobant V Bousquet-Dubouch M-P Colau D Chapiro J Parmentier N. Analysis of the Processing of Seven Human Tumor Antigens by Intermediate Proteasomes. J Immunol (2012) 189:3538–47. doi: 10.4049/JIMMUNOL.1103213 22925930

[118] Morel S Lévy F Burlet-Schiltz O Brasseur F Probst-Kepper M Peitrequin A. Processing of Some Antigens by the Standard Proteasome But Not by the Immunoproteasome Results in Poor Presentation by Dendritic Cells. Immunity (2000) 12:107–17. doi: 10.1016/S1074-7613(00)80163-6 10661410

[119] Chapiro J Claverol S Piette F Ma W Stroobant V Guillaume B. Destructive Cleavage of Antigenic Peptides Either by the Immunoproteasome or by the Standard Proteasome Results in Differential Antigen Presentation. J Immunol (2006) 176:1053–61. doi: 10.4049/JIMMUNOL.176.2.1053 16393993

[120] Kalaora S Lee JS Barnea E Levy R Greenberg P Alon M. Immunoproteasome Expression is Associated With Better Prognosis and Response to Checkpoint Therapies in Melanoma. Nat Commun (2020) 11:1–12. doi: 10.1038/s41467-020-14639-9 32060274 PMC7021791

[121] Greenberg PD. Selecting and Validating Targets, Isolating High Affinity TCRs, and Engineering T Cells That Can Be Effective in Therapy, in: 2nd NCI Workshop on Cell-Based Immunotherapy for Solid Tumors . Available at: https://nci.rev.vbrick.com/ (Accessed October 20, 2021).

[122] Liu Q Hao C Su P Shi J. Down-Regulation of HLA Class I Antigen-Processing Machinery Components in Esophageal Squamous Cell Carcinomas: Association With Disease Progression. Scand J Gastroenterol (2009) 44:960–9. doi: 10.1080/00365520902998679 19492245

[123] Hasim A Abudula M Aimiduo R Ma J-Q Jiao Z. Post-Transcriptional and Epigenetic Regulation of Antigen Processing Machinery (APM) Components and HLA-I in Cervical Cancers From Uighur Women. PloS One (2012) 7:e44952. doi: 10.1371/journal.pone.0044952 23024775 PMC3443204

[124] Parkhurst MR Yang JC Langan RC Dudley ME Nathan D-AN Feldman SA. T Cells Targeting Carcinoembryonic Antigen Can Mediate Regression of Metastatic Colorectal Cancer But Induce Severe Transient Colitis. Mol Ther (2011) 19:620–6. doi: 10.1038/MT.2010.272 PMC3048186 21157437

[125] Yee C Thompson JA Roche P Byrd DR Lee PP Piepkorn M. Melanocyte Destruction After Antigen-Specific Immunotherapy of Melanoma: Direct Evidence of T Cell–mediated Vitiligo. J Exp Med (2000) 192:1637–44. doi: 10.1084/JEM.192.11.1637 PMC2193107 11104805

[126] Johnson LA Morgan RA Dudley ME Cassard L Yang JC Hughes MS. Gene Therapy With Human and Mouse T-Cell Receptors Mediates Cancer Regression and Targets Normal Tissues Expressing Cognate Antigen. Blood (2009) 114:535–46. doi: 10.1182/BLOOD-2009-03-211714 PMC2929689 19451549

[127] Cameron BJ Gerry AB Dukes J Harper JV Kannan V Bianchi FC. Identification of a Titin-Derived HLA-A1–Presented Peptide as a Cross-Reactive Target for Engineered MAGE A3–Directed T Cells. Sci Transl Med (2013) 5:197ra103. doi: 10.1126/SCITRANSLMED.3006034 PMC6002776 23926201

[128] Linette GP Stadtmauer EA Maus MV Rapoport AP Levine BL Emery L. Cardiovascular Toxicity and Titin Cross-Reactivity of Affinity-Enhanced T Cells in Myeloma and Melanoma. Blood (2013) 122:863–71. doi: 10.1182/BLOOD-2013-03-490565 PMC3743463 23770775

[129] Morgan R Chinnasamy N Abate-Daga D Gros A Robbins P Zheng Z. Cancer Regression and Neurological Toxicity Following Anti-MAGE-A3 TCR Gene Therapy. J Immunother (2013) 36:133–51. doi: 10.1097/CJI.0B013E3182829903 PMC3581823 23377668

[130] Almeida LG Sakabe NJ de Oliveira AR Silva MCC Mundstein AS Cohen T. CTdatabase: A Knowledge-Base of High-Throughput and Curated Data on Cancer-Testis Antigens. Nucleic Acids Res (2009) 37:D816–9. doi: 10.1093/NAR/GKN673 PMC2686577 18838390

[131] Kunert A Obenaus M Lamers C Blankenstein T Debets R. T-Cell Receptors for Clinical Therapy: *In Vitro* Assessment of Toxicity Risk. Clin Cancer Res (2017) 23:6012–20. doi: 10.1158/1078-0432.CCR-17-1012 28645940

[132] Luo X Cui H Cai L Zhu W Yang W Patrick M. Selection of a Clinical Lead TCR Targeting Alpha-Fetoprotein-Positive Liver Cancer Based on a Balance of Risk and Benefit. Front Immunol (2020) 11:623. doi: 10.3389/FIMMU.2020.00623 32425926 PMC7203609

[133] Sanderson J Crowley D Wiedermann G Quinn L Crossland K Tunbridge H. Preclinical Evaluation of an Affinity-Enhanced MAGE-A4-Specific T-Cell Receptor for Adoptive T-Cell Therapy. Oncoimmunology (2019) 9:e1682381. doi: 10.1080/2162402X.2019.1682381 PMC6959444 32002290

[134] Cai L Caraballo Galva LD Peng Y Luo X Zhu W Yao Y. Preclinical Studies of the Off-Target Reactivity of AFP158-Specific TCR Engineered T Cells. Front Immunol (2020) 11:607. doi: 10.3389/FIMMU.2020.00607 32395117 PMC7196607

[135] Stone JD Kranz DM. Role of T Cell Receptor Affinity in the Efficacy and Specificity of Adoptive T Cell Therapies. Front Immunol (2013) 4:244. doi: 10.3389/FIMMU.2013.00244 23970885 PMC3748443

[136] Minami Y Weissman AM Samelson LE Klausner RD. Building a Multichain Receptor: Synthesis, Degradation, and Assembly of the T-Cell Antigen Receptor. Biochemistry (1987) 84:2688–92. doi: 10.1073/pnas.84.9.2688 PMC304723 3495001

[137] Bendle GM Linnemann C Hooijkaas AI Bies L de Witte MA Jorritsma A. Lethal Graft-Versus-Host Disease in Mouse Models of T Cell Receptor Gene Therapy. Nat Med (2010) 16:565–70. doi: 10.1038/nm.2128 20400962

[138] van Loenen MM de BR AL A RS H GL V Willemze R. Mixed T Cell Receptor Dimers Harbor Potentially Harmful Neoreactivity. Proc Natl Acad Sci (2010) 107:10972–7. doi: 10.1073/PNAS.1005802107 PMC2890759 20534461

[139] Rosenberg SA. Of Mice, Not Men: No Evidence for Graft-Versus-Host Disease in Humans Receiving T-Cell Receptor–Transduced Autologous T Cells. Mol Ther (2010) 18:1744–5. doi: 10.1038/MT.2010.195 PMC2951571 20885433

[140] Cohen CJ Zhao Y Zheng Z Rosenberg SA Morgan RA. Enhanced Antitumor Activity of Murine-Human Hybrid T-Cell Receptor (TCR) in Human Lymphocytes Is Associated With Improved Pairing and TCR/CD3 Stability. Cancer Res (2006) 66:8878–86. doi: 10.1158/0008-5472.CAN-06-1450 PMC2147082 16951205

[141] Stripecke R Villacres C Skelton DC Satake N Halene S Kohn DB. Immune Response to Green Fluorescent Protein: Implications for Gene Therapy. Gene Ther (1999) 6:1305–12. doi: 10.1038/sj.gt.3300951 10455440

[142] Rosenzweig M Connole M Glickman R Yue S-PS Noren B DeMaria M. Induction of Cytotoxic T Lymphocyte and Antibody Responses to Enhanced Green Fluorescent Protein Following Transplantation of Transduced CD34+ Hematopoietic Cells. Blood (2001) 97:1951–9. doi: 10.1182/BLOOD.V97.7.1951 11264158

[143] Riddell SR Elliott M Lewinsohn DA Gilbert MJ Wilson L Manley SA. T-Cell Mediated Rejection of Gene-Modified HIV-Specific Cytotoxic T Lymphocytes in HIV-Infected Patients. Nat Med (1996) 2:216–23. doi: 10.1038/nm0296-216 8574968

[144] Davis J Theoret M Zheng Z Lamers C Rosenberg S Morgan R. Development of Human Anti-Murine T-Cell Receptor Antibodies in Both Responding and Nonresponding Patients Enrolled in TCR Gene Therapy Trials. Clin Cancer Res (2010) 16:5852–61. doi: 10.1158/1078-0432.CCR-10-1280 PMC3058233 21138872

[145] Bialer G Horovitz-Fried M Ya’acobi S Morgan RA Cohen CJ. Selected Murine Residues Endow Human TCR With Enhanced Tumor Recognition. J Immunol (2010) 184:6232–41. doi: 10.4049/JIMMUNOL.0902047 20427762

[146] Sommermeyer D Uckert W. Minimal Amino Acid Exchange in Human TCR Constant Regions Fosters Improved Function of TCR Gene-Modified T Cells. J Immunol (2010) 184:6223–31. doi: 10.4049/JIMMUNOL.0902055 20483785

[147] Krshnan L Park S Im W Call MJ Call ME. A Conserved αβ Transmembrane Interface Forms the Core of a Compact T-Cell Receptor–CD3 Structure Within the Membrane. Proc Natl Acad Sci USA (2016) 113:E6649. doi: 10.1073/PNAS.1611445113 27791034 PMC5086997

[148] Kuball J Dossett ML Wolfl M Ho WY Voss R-H Fowler C. Facilitating Matched Pairing and Expression of TCR Chains Introduced Into Human T Cells. Blood (2007) 109:2331–8. doi: 10.1182/BLOOD-2006-05-023069 PMC1852191 17082316

[149] Cohen CJ Li YF El-Gamil M Robbins PF Rosenberg SA Morgan RA. Enhanced Antitumor Activity of T Cells Engineered to Express T-Cell Receptors With a Second Disulfide Bond. Cancer Res (2007) 67:3898–903. doi: 10.1158/0008-5472.CAN-06-3986 PMC2147081 17440104

[150] Haga-Friedman A Horovitz-Fried M Cohen CJ. Incorporation of Transmembrane Hydrophobic Mutations in the TCR Enhance Its Surface Expression and T Cell Functional Avidity. J Immunol (2012) 188:5538–46. doi: 10.4049/JIMMUNOL.1103020 22544927

[151] Voss R-H Willemsen RA Kuball J Grabowski M Engel R Intan RS. Molecular Design of the Cαβ Interface Favors Specific Pairing of Introduced Tcrαβ in Human T Cells. J Immunol (2008) 180:391–401. doi: 10.4049/JIMMUNOL.180.1.391 18097040

[152] Bethune MT Gee MH Bunse M Lee MS Gschweng EH Pagadala MS. Domain-Swapped T Cell Receptors Improve the Safety of TCR Gene Therapy. Elife (2016) 5:e19095. doi: 10.7554/ELIFE.19095 27823582 PMC5101000

[153] Willemsen RA Weijtens MEM Ronteltap C Eshhar Z Gratama JW Chames P. Grafting Primary Human T Lymphocytes With Cancer-Specific Chimeric Single Chain and Two Chain TCR. Gene Ther (2000) 7:1369–77. doi: 10.1038/sj.gt.3301253 10981663

[154] Sebestyén Z Schooten E Sals T Zaldivar I José ES Alarcón B. Human TCR That Incorporate Cd3ζ Induce Highly Preferred Pairing Between Tcrα and β Chains Following Gene Transfer. J Immunol (2008) 180:7736–46. doi: 10.4049/JIMMUNOL.180.11.7736 18490778

[155] Govers C Sebestyén Z Roszik J van Brakel M Berrevoets C Szöőr Á. TCRs Genetically Linked to CD28 and CD3ϵ Do Not Mispair With Endogenous TCR Chains and Mediate Enhanced T Cell Persistence and Anti-Melanoma Activity. J Immunol (2014) 193:5315–26. doi: 10.4049/jimmunol.1302074 25320284

[156] Willcoj BE Gao GF Wyer JR O’callaghan CA Jakobsen BK Boulter JM. Production of Soluble αβ T-Cell Receptor Heterodimers Suitable for Biophysical Analysis of Ligand Binding. Protein Sci (2008) 8:2418–23. doi: 10.1110/PS.8.11.2418 PMC2144200 10595544

[157] Foley KC Spear TT Murray DC Nagato K Garrett-Mayer E Nishimura MI. HCV T Cell Receptor Chain Modifications to Enhance Expression, Pairing, and Antigen Recognition in T Cells for Adoptive Transfer. Mol Ther - Oncolytics (2017) 5:105–15. doi: 10.1016/J.OMTO.2017.05.004 PMC5447397 28573185

[158] Chung S Wucherpfennig KW Friedman SM Hafler DA Strominger JL. Functional Three-Domain Single-Chain T-Cell Receptors. Proc Natl Acad Sci U.S.A. (1994) 91:12654–8. doi: 10.1073/PNAS.91.26.12654 PMC45497 7809095

[159] Plaksin D Polakova K McPhie P Margulies DH. A Three-Domain T Cell Receptor is Biologically Active and Specifically Stains Cell Surface MHC/peptide Complexes. J Immunol (1997) 158:2218–27. 9036968

[160] Zhang T He X Tsang TC Harris DT. Transgenic TCR Expression: Comparison of Single Chain With Full-Length Receptor Constructs for T-Cell Function. Cancer Gene Ther (2004) 11:487–96. doi: 10.1038/sj.cgt.7700703 15153936

[161] Voss R-H Thomas S Pfirschke C Hauptrock B Klobuch S Kuball J. Coexpression of the T-Cell Receptor Constant α Domain Triggers Tumor Reactivity of Single-Chain TCR-Transduced Human T Cells. Blood (2010) 115:5154–63. doi: 10.1182/BLOOD-2009-11-254078 20378753

[162] Aggen DH Chervin AS Schmitt TM Engels B Stone JD Richman SA. Single-Chain Vαvβ T-Cell Receptors Function Without Mispairing With Endogenous TCR Chains. Gene Ther (2011) 19:365–74. doi: 10.1038/gt.2011.104 PMC3321103 21753797

[163] Provasi E Genovese P Lombardo A Magnani Z Liu P-Q Reik A. Editing T Cell Specificity Towards Leukemia by Zinc-Finger Nucleases and Lentiviral Gene Transfer. Nat Med (2012) 18:807. doi: 10.1038/NM.2700 22466705 PMC5019824

[164] Stadtmauer EA Fraietta JA Davis MM Cohen AD Weber KL Lancaster E. CRISPR-Engineered T Cells in Patients With Refractory Cancer. Science (2020) 367:eaba7365. doi: 10.1126/SCIENCE.ABA7365 32029687 PMC11249135

[165] Campillo-Davo D Fujiki F Van den Bergh JMJ De Reu H Smits ELJM Goossens H. Efficient and Non-Genotoxic RNA-Based Engineering of Human T Cells Using Tumor-Specific T Cell Receptors With Minimal TCR Mispairing. Front Immunol (2018) 9:2503. doi: 10.3389/FIMMU.2018.02503 30464762 PMC6234959

[166] Roth TL Puig-Saus C Yu R Shifrut E Carnevale J Li PJ. Reprogramming Human T Cell Function and Specificity With Non-Viral Genome Targeting. Nature (2018) 559:405. doi: 10.1038/S41586-018-0326-5 29995861 PMC6239417

[167] Li Y Moysey R Molloy PE Vuidepot AL Mahon T Baston E. Directed Evolution of Human T-Cell Receptors With Picomolar Affinities by Phage Display. Nat Biotechnol (2005) 23:349–54. doi: 10.1038/nbt1070 15723046

[168] Robbins PF Li YF El-Gamil M Zhao Y Wargo JA Zheng Z. Single and Dual Amino Acid Substitutions in TCR CDRs Can Enhance Antigen-Specific T Cell Functions. J Immunol (2008) 180:6116–31. doi: 10.4049/jimmunol.180.9.6116 PMC2424230 18424733

[169] Holler PD Holman PO Shusta EV O’Herrin S Wittrup KD Kranz DM. *In Vitro* Evolution of a T Cell Receptor With High Affinity for Peptide/MHC. Proc Natl Acad Sci USA (2000) 97:5387–92. doi: 10.1073/pnas.080078297 PMC25838 10779548

[170] Hellman LM Foley KC Singh NK Alonso JA Riley TP Devlin JR. Improving T Cell Receptor On-Target Specificity *via* Structure-Guided Design. Mol Ther (2019) 27:300–13. doi: 10.1016/j.ymthe.2018.12.010 PMC6369632 30617019

[171] Morgan RA Dudley ME Wunderlich JR Hughes MS Yang JC Sherry RM. Cancer Regression in Patients After Transfer of Genetically Engineered Lymphocytes. Science (2006) 314:126–9. doi: 10.1126/SCIENCE.1129003 PMC2267026 16946036

[172] Chodon T Comin-Anduix B Chmielowski B Koya RC Wu Z Auerbach M. Adoptive Transfer of MART-1 T-Cell Receptor Transgenic Lymphocytes and Dendritic Cell Vaccination in Patients With Metastatic Melanoma. Clin Cancer Res (2014) 20:2457–65. doi: 10.1158/1078-0432.CCR-13-3017 PMC4070853 24634374

[173] Moore T Wagner C Scurti G Hutchens K Godellas C Clark A. Clinical and Immunologic evaluation of Three Metastatic Melanoma Patients Treated With Autologous Melanoma-Reactive TCR-Transduced T Cells. Cancer Immunol Immunother (2018) 67:311–25. doi: 10.1007/S00262-017-2073-0 PMC5935006 29052782

[174] Robbins PF Kassim SH Tran TLN Crystal JS Morgan RA Feldman SA. A Pilot Trial Using Lymphocytes Genetically Engineered With an NY-ESO-1–Reactive T-Cell Receptor: Long-Term Follow-Up and Correlates With Response. Clin Cancer Res (2015) 21:1019–27. doi: 10.1158/1078-0432.CCR-14-2708 PMC4361810 25538264

[175] Ishihara M Kitano S Hattori H Miyahara Y Kato H Mishima H. Tumor Responses and Early Onset Cytokine Release Syndrome in Synovial Sarcoma Patients Treated With a Novel Affinity-Enhanced NY-ESO-1-Targeting TCR-Redirected T Cell Transfer [Conference presentation]. Am Soc Clin Oncol (2019) 530. doi: 10.1200/JCO.2019.37.15_SUPPL.2530

[176] Nowicki T Berent-Maoz B Cheung-Lau G Huang R Wang X Tsoi J. A Pilot Trial of the Combination of Transgenic NY-ESO-1-Reactive Adoptive Cellular Therapy With Dendritic Cell Vaccination With or Without Ipilimumab. Clin Cancer Res (2019) 25:2096–108. doi: 10.1158/1078-0432.CCR-18-3496 PMC6445780 30573690

[177] Butler MO Sotov V Saibil S Bonilla L Boross-Harmer S Fyrsta M. Adoptive T Cell Therapy With TBI-1301 Results in Gene-Engineered T Cell Persistence and Anti-Tumour Responses in Patients With NY-ESO-1 Expressing Solid Tumours. Ann Oncol (2019) 30:v481. doi: 10.1093/ANNONC/MDZ253.009

[178] Stadtmauer E Faitg T Lowther D Badros A Chagin K Dengel K. Long-Term Safety and Activity of NY-ESO-1 SPEAR T Cells After Autologous Stem Cell Transplant for Myeloma. Blood Adv (2019) 3:2022–34. doi: 10.1182/BLOODADVANCES.2019000194 PMC6616263 31289029

[179] Ramachandran I Lowther D Dryer-Minnerly R Wang R Fayngerts S Nunez D. Systemic and Local Immunity Following Adoptive Transfer of NY-ESO-1 SPEAR T Cells in Synovial Sarcoma. J Immunother Cancer (2019) 7:276. doi: 10.1186/S40425-019-0762-2 31651363 PMC6813983

[180] Kageyama S Ikeda H Miyahara Y Imai N Ishihara M Saito K. Adoptive Transfer of MAGE-A4 T-Cell Receptor Gene-Transduced Lymphocytes in Patients With Recurrent Esophageal Cancer. Clin Cancer Res (2015) 21:2268–77. doi: 10.1158/1078-0432.CCR-14-1559 25855804

[181] Berard M Brandt K Bulfone-Paus S Tough DF. IL-15 Promotes the Survival of Naive and Memory Phenotype CD8 + T Cells. J Immunol (2003) 170:5018–26. doi: 10.4049/jimmunol.170.10.5018 12734346

[182] Hong DS Van Tine BA Olszanski AJ Johnson ML Liebner DA Trivedi T. Phase I Dose Escalation and Expansion Trial to Assess the Safety and Efficacy of ADP-A2M4 SPEAR T Cells in Advanced Solid Tumors [Conference Presentation]. In: 2020 ASCO Annual Meeting (American Society of Clinical Oncology). Journal of Clinical Immunology (2020) p. 102. doi: 10.1200/JCO.2020.38.15_SUPPL.102

[183] Hong D Clarke J Johanns T Kebriaei P Heymach J Galal A. Initial Safety, Efficacy, and Product Attributes From the SURPASS Trial With ADP-A2M4CD8, a SPEAR T-Cell Therapy Incorporating an Affinity Optimized TCR Targeting MAGE-A4 and a CD8α Co-Receptor [Conference Presentation]. In: SITC 2020 (BMJ Specialist Journals). Journal for ImmunoTherapy of Cancer (2020) p. A231. doi: 10.1136/JITC-2020-SITC2020.0379

[184] D’Angelo SP Van Tine BA Attia S Blay J-Y Strauss SJ Morales CMV. SPEARHEAD-1: A Phase 2 Trial of Afamitresgene Autoleucel (Formerly ADP-A2M4) in Patients With Advanced Synovial Sarcoma or Myxoid/Round Cell Liposarcoma [Conference Presentation]. In: 2021 ASCO Annual Meeting (Wolters Kluwer Health). Journal of Clinical Immunology (2020) p. 11504–4. doi: 10.1200/JCO.2021.39.15_SUPPL.11504

[185] Lam VK Hong DS Heymach J Blumenschein GR Creelan BC Bradbury PA. Initial Safety Assessment of MAGE-A10c796TCR T-Cells in Two Clinical Trials [Conference presentation]. Am Soc Clin Oncol (2018) 3056. doi: 10.1200/JCO.2018.36.15_SUPPL.3056

[186] Doran SL Stevanović S Adhikary S Gartner JJ Jia L Kwong MLM. T-Cell Receptor Gene Therapy for Human Papillomavirus–Associated Epithelial Cancers: A First-In-Human, Phase I/II Study. J Clin Oncol (2019) 37:2759–68. doi: 10.1200/JCO.18.02424 PMC6800280 31408414

[187] Tawara I Kageyama S Miyahara Y Fujiwara H Nishida T Akatsuka Y. Safety and Persistence of WT1-Specific T-Cell Receptor Gene–Transduced Lymphocytes in Patients With AML and MDS. Blood (2017) 130:1985–94. doi: 10.1182/BLOOD-2017-06-791202 28860210

[188] Chapuis AG Egan DN Bar M Schmitt TM McAfee MS Paulson KG. T Cell Receptor Gene Therapy Targeting WT1 Prevents Acute Myeloid Leukemia Relapse Post-Transplant. Nat Med (2019) 25:1064–72. doi: 10.1038/s41591-019-0472-9 PMC6982533 31235963

[189] Bechman N Maher J. Lymphodepletion Strategies to Potentiate Adoptive T-Cell Immunotherapy–What are We Doing; Where are We Going? Expert Opin Biol Ther (2021) 21:627–37. doi: 10.1080/14712598.2021.1857361 33243003

[190] Jiang T Zhou C Ren S. Role of IL-2 in Cancer Immunotherapy. Oncoimmunology (2016) 5:e1163462. doi: 10.1080/2162402X.2016.1163462 27471638 PMC4938354

[191] Rosenberg SA. IL-2: The First Effective Immunotherapy for Human Cancer. J Immunol (2014) 192:5451–8. doi: 10.4049/JIMMUNOL.1490019 PMC6293462 24907378

[192] D’Angelo S Melchiori L Merchant M Bernstein D Glod J Kaplan R. Antitumor Activity Associated With Prolonged Persistence of Adoptively Transferred NY-ESO-1 C259 T Cells in Synovial Sarcoma. Cancer Discov (2018) 8:944–57. doi: 10.1158/2159-8290.CD-17-1417 PMC8092079 29891538

[193] Ishihara M Kitano S Hattori H Miyahara Y Kato H Mishima H. Tumor Responses and Early Onset Cytokine Release Syndrome in Synovial Sarcoma Patients Treated With a Novel Affinity-Enhanced NY-ESO-1-Targeting TCR-Redirected T Cell Transfer [Conference Presentation]. In: ASCO Annual Meeting (American Society of Clinical Oncology). (2019). p. 2530. doi: 10.1200/JCO.2019.37.15_SUPPL.2530

[194] Lu Y Parker L Lu T Zheng Z Toomey M White D. Treatment of Patients With Metastatic Cancer Using a Major Histocompatibility Complex Class II-Restricted T-Cell Receptor Targeting the Cancer Germline Antigen MAGE-A3. J Clin Oncol (2017) 35:3322–9. doi: 10.1200/JCO.2017.74.5463 PMC5652397 28809608

[195] Wagner J Wickman E Derenzo C Gottschalk S. CAR T Cell Therapy for Solid Tumors: Bright Future or Dark Reality? Mol Ther (2020) 28:2320–39. doi: 10.1016/j.ymthe.2020.09.015 PMC7647674 32979309

[196] Calderon H Mamonkin M Guedan S. Analysis of CAR-Mediated Tonic Signaling. Methods Mol Biol (2020) 2086:223–36. doi: 10.1007/978-1-0716-0146-4_17 31707680

[197] Ajina A Maher J. Strategies to Address Chimeric Antigen Receptor Tonic Signalling. Mol Cancer Ther (2018) 17:1795–815. doi: 10.1158/1535-7163.MCT-17-1097 PMC6130819 30181329

[198] Fiorenza S Ritchie DS Ramsey SD Turtle CJ Roth JA. Value and Affordability of CAR T-Cell Therapy in the United States. Bone Marrow Transplant (2020) 55:1706–15. doi: 10.1038/s41409-020-0956-8 32474570

[199] Tzannou I Papadopoulou A Naik S Leung K Martinez CA Ramos CA. Off-The-Shelf Virus-Specific T Cells to Treat BK Virus, Human Herpesvirus 6, Cytomegalovirus, Epstein-Barr Virus, and Adenovirus Infections After Allogeneic Hematopoietic Stem-Cell Transplantation. J Clin Oncol (2017) 35:3547–57. doi: 10.1200/JCO.2017.73.0655 PMC5662844 28783452

[200] Nussbaumer O Koslowski M. The Emerging Role of γδ T Cells in Cancer Immunotherapy. Immuno-Oncol Technol (2019) 1:3–10. doi: 10.1016/j.iotech.2019.06.002 PMC9216673 35755322

[201] Heczey A Liu D Tian G Courtney AN Wei J Marinova E. Invariant NKT Cells With Chimeric Antigen Receptor Provide a Novel Platform for Safe and Effective Cancer Immunotherapy. Blood (2014) 124:2824. doi: 10.1182/BLOOD-2013-11-541235 25049283 PMC4215313

[202] Ren J Liu X Fang C Jiang S June CH Zhao Y. Multiplex Genome Editing to Generate Universal CAR T Cells Resistant to PD1 Inhibition. Clin Cancer Res (2017) 23:2255–66. doi: 10.1158/1078-0432.CCR-16-1300 PMC5413401 27815355

[203] Zhao Y Zheng Z Cohen CJ Gattinoni L Palmer DC Restifo NP. High-Efficiency Transfection of Primary Human and Mouse T Lymphocytes Using RNA Electroporation. Mol Ther (2006) 13:151–9. doi: 10.1016/J.YMTHE.2005.07.688 PMC1473967 16140584

[204] Birkholz K Hombach A Krug C Reuter S Kershaw M Kämpgen E. Transfer of mRNA Encoding Recombinant Immunoreceptors Reprograms CD4+ and CD8+ T Cells for Use in the Adoptive Immunotherapy of Cancer. Gene Ther (2009) 16:596–604. doi: 10.1038/gt.2008.189 19158846

[205] Nakazawa Y Huye LE Dotti G Foster AE Vera JF Manuri PR. Optimization of the PiggyBac Transposon System for the Sustained Genetic Modification of Human T-Lymphocytes. J Immunother (2009) 32:826–36. doi: 10.1097/CJI.0B013E3181AD762B PMC2796278 19752751

[206] Deniger DC Pasetto A Tran E Parkhurst MR Cohen CJ Robbins PF. Stable, Nonviral Expression of Mutated Tumor Neoantigen-Specific T-Cell Receptors Using the Sleeping Beauty Transposon/Transposase System. Mol Ther (2016) 24:1078–89. doi: 10.1038/MT.2016.51 PMC4923320 26945006

[207] Morita D Nishio N Saito S Tanaka M Kawashima N Okuno Y. Enhanced Expression of Anti-CD19 Chimeric Antigen Receptor in Piggybac Transposon-Engineered T Cells. Mol Ther - Methods Clin Dev (2018) 8:131–40. doi: 10.1016/J.OMTM.2017.12.003 PMC5907825 29687032

[208] Chan JD Lai J Slaney CY Kallies A Beavis PA Darcy PK. Cellular Networks Controlling T Cell Persistence in Adoptive Cell Therapy. Nat Rev Immunol (2021) 21:769–84. doi: 10.1038/s41577-021-00539-6 33879873

[209] Gattinoni L Klebanoff CA Palmer DC Wrzesinski C Kerstann K Yu Z. Acquisition of Full Effector Function *In Vitro* Paradoxically Impairs the *In Vivo* Antitumor Efficacy of Adoptively Transferred CD8+ T Cells. J Clin Invest (2005) 115:1616–26. doi: 10.1172/JCI24480 PMC1137001 15931392

[210] Klebanoff CA Gattinoni L Torabi-Parizi P Kerstann K Cardones AR Finkelstein SE. Central Memory Self/Tumor-Reactive CD8+ T Cells Confer Superior Antitumor Immunity Compared With Effector Memory T Cells. Proc Natl Acad Sci (2005) 102:9571–6. doi: 10.1073/PNAS.0503726102 PMC1172264 15980149

[211] Gattinoni L Lugli E Ji Y Pos Z Paulos CM Quigley MF. A Human Memory T-Cell Subset With Stem Cell-Like Properties. Nat Med (2011) 17:1290–7. doi: 10.1038/NM.2446 PMC3192229 21926977

[212] Klebanoff CA Finkelstein SE Surman DR Lichtman MK Gattinoni L Theoret MR. IL-15 Enhances the *In Vivo* Antitumor Activity of Tumor-Reactive CD8+ T Cells. Proc Natl Acad Sci USA (2004) 101:1969–74. doi: 10.1073/PNAS.0307298101 PMC357036 14762166

[213] Mo F Yu Z Li P Oh J Spolski R Zhao L. An Engineered IL-2 Partial Agonist Promotes CD8+ T Cell Stemness. Nature (2021) 597:544–8. doi: 10.1038/s41586-021-03861-0 PMC9172917 34526724

[214] Nalawade S Shafer P Bajgain P McKenna K Ali A Kelly L. Selectively Targeting Myeloid-Derived Suppressor Cells Through TRAIL Receptor 2 to Enhance the Efficacy of CAR T Cell Therapy for Treatment of Breast Cancer. J Immunother Cancer (2021) 9:e003237. doi: 10.1136/jitc-2021-003237 34815355 PMC8611441

[215] Oda S Daman A Garcia N Wagener F Schmitt T Tan X. A CD200R-CD28 Fusion Protein Appropriates an Inhibitory Signal to Enhance T-Cell Function and Therapy of Murine Leukemia. Blood (2017) 130:2410–9. doi: 10.1182/BLOOD-2017-04-777052 PMC5709784 29042364

[216] Feng J Xu H Cinquina A Wu Z Chen Q Zhang P. Treatment of Aggressive T Cell Lymphoblastic Lymphoma/leukemia Using Anti-CD5 CAR T Cells. Stem Cell Rev Rep (2021) 17:652–61. doi: 10.1007/S12015-020-10092-9 PMC8036178 33410096

[217] Pegram HJ Lee JC Hayman EG Imperato GH Tedder TF Sadelain M. Tumor-Targeted T Cells Modified to Secrete IL-12 Eradicate Systemic Tumors Without Need for Prior Conditioning. Blood (2012) 119:4133–41. doi: 10.1182/BLOOD-2011-12-400044 PMC3359735 22354001

[218] Koneru M Purdon TJ Spriggs D Koneru S Brentjens RJ. IL-12 Secreting Tumor-Targeted Chimeric Antigen Receptor T Cells Eradicate Ovarian Tumors *In Vivo* . Oncoimmunology (2015) 4:e994446. doi: 10.4161/2162402X.2014.994446 25949921 PMC4404840

[219] Hoyos V Savoldo B Quintarelli C Mahendravada A Zhang M Vera J. Engineering CD19-Specific T Lymphocytes With Interleukin-15 and a Suicide Gene to Enhance Their Anti-Lymphoma/Leukemia Effects and Safety. Leukemia (2010) 24:1160–70. doi: 10.1038/LEU.2010.75 PMC2888148 20428207

[220] Shum T Omer B Tashiro H Kruse RL Wagner DL Parikh K. Constitutive Signaling From an Engineered IL7 Receptor Promotes Durable Tumor Elimination by Tumor-Redirected T Cells. Cancer Discov (2017) 7:1238–47. doi: 10.1158/2159-8290.CD-17-0538 PMC5669830 28830878

[221] Ma S Li X Wang X Cheng L Li Z Zhang C. Current Progress in Car-T Cell Therapy for Solid Tumors. Int J Biol Sci (2019) 15:2548–60. doi: 10.7150/ijbs.34213 PMC6854376 31754328

[222] Irving M de Silly RV Scholten K Dilek N Coukos G. Engineering Chimeric Antigen Receptor T-Cells for Racing in Solid Tumors: Don’t Forget the Fuel. Front Immunol (2017) 8:267. doi: 10.3389/fimmu.2017.00267 28421069 PMC5376574

[223] Martinez M Moon EK. CAR T Cells for Solid Tumors: New Strategies for Finding, Infiltrating, and Surviving in the Tumor Microenvironment. Front Immunol (2019) 10:128. doi: 10.3389/fimmu.2019.00128 30804938 PMC6370640

[224] Bajgain P Tawinwung S D’Elia L Sukumaran S Watanabe N Hoyos V. CAR T Cell Therapy for Breast Cancer: Harnessing the Tumor Milieu to Drive T Cell Activation. J Immunother Cancer (2018) 6:1–13. doi: 10.1186/s40425-018-0347-5 29747685 PMC5944113

[225] Corbière V Chapiro J Stroobant V Ma W Lurquin C Lethé B. Antigen Spreading Contributes to MAGE Vaccination-Induced Regression of Melanoma Metastases. Cancer Res (2011) 71:1253–62. doi: 10.1158/0008-5472.CAN-10-2693 21216894

[226] Chapuis AG Roberts IM Thompson JA Margolin KA Bhatia S Lee SM. T-Cell Therapy Using Interleukin-21–Primed Cytotoxic T-Cell Lymphocytes Combined With Cytotoxic T-Cell Lymphocyte Antigen-4 Blockade Results in Long-Term Cell Persistence and Durable Tumor Regression. J Clin Oncol (2016) 34:3787–95. doi: 10.1200/JCO.2015.65.5142 PMC5477923 27269940

[227] Vasileiou S Lulla PD Tzannou I Watanabe A Kuvalekar M Callejas WL. T-Cell Therapy for Lymphoma Using Nonengineered Multiantigen-Targeted T Cells Is Safe and Produces Durable Clinical Effects. J Clin Oncol (2021) 39:1415–25. doi: 10.1200/JCO.20.02224 PMC8274795 33507803

[228] Avanzi MP Yeku O Li X Wijewarnasuriya DP van Leeuwen DG Cheung K. Engineered Tumor-Targeted T Cells Mediate Enhanced Anti-Tumor Efficacy Both Directly and Through Activation of the Endogenous Immune System. Cell Rep (2018) 23:2130–41. doi: 10.1016/J.CELREP.2018.04.051 PMC5986286 29768210

[229] Curran KJ Seinstra BA Nikhamin Y Yeh R Usachenko Y Van Leeuwen DG. Enhancing Antitumor Efficacy of Chimeric Antigen Receptor T Cells Through Constitutive CD40L Expression. Mol Ther (2015) 23:769–78. doi: 10.1038/MT.2015.4 PMC4395796 25582824

[230] Lai J Mardiana S House IG Sek K Henderson MA Giuffrida L. Adoptive Cellular Therapy With T Cells Expressing the Dendritic Cell Growth Factor Flt3L Drives Epitope Spreading and Antitumor Immunity. Nat Immunol (2020) 21:914–26. doi: 10.1038/s41590-020-0676-7 32424363

[231] Shah NN Maatman T Hari P Johnson B. Multi Targeted CAR-T Cell Therapies for B-Cell Malignancies. Front Oncol (2019) 9:146. doi: 10.3389/FONC.2019.00146 30915277 PMC6423158

[232] Jhunjhunwala S Hammer C Delamarre L. Antigen Presentation in Cancer: Insights Into Tumour Immunogenicity and Immune Evasion. Nat Rev Cancer (2021) 21:298–312. doi: 10.1038/s41568-021-00339-z 33750922

[233] Dhatchinamoorthy K Colbert JD Rock KL. Cancer Immune Evasion Through Loss of MHC Class I Antigen Presentation. Front Immunol (2021) 12:636568. doi: 10.3389/fimmu.2021.636568 33767702 PMC7986854

[234] Propper DJ Chao D Braybrooke JP Bahl P Thavasu P Balkwill F. Low-Dose IFN-γ Induces Tumor MHC Expression in Metastatic Malignant Melanoma. Clin Cancer Res (2003) 9:84–92. 12538455

[235] Vlková V Štěpánek I Hrušková V Šenigl F Mayerová V Šrámek M. Epigenetic Regulations in the Ifnγ Signalling Pathway: Ifnγ-Mediated MHC Class I Upregulation on Tumour Cells Is Associated With DNA Demethylation of Antigen-Presenting Machinery Genes. Oncotarget (2014) 5:6923. doi: 10.18632/ONCOTARGET.2222 25071011 PMC4196173

[236] Del Campo AB Aptsiauri N Méndez R Zinchenko S Vales A Paschen A. Efficient Recovery of HLA Class I Expression in Human Tumor Cells After Beta2-Microglobulin Gene Transfer Using Adenoviral Vector: Implications for Cancer Immunotherapy. Scand J Immunol (2009) 70:125–35. doi: 10.1111/J.1365-3083.2009.02276.X 19630918

[237] Hiscox MJ Wasmuth A Williams CL Foot JN Wiedermann GE Fadda V. In Vitro Selection and Engineering of a Human Leukocyte Antigen-Independent T-Cell Receptor Recognizing Human Mesothelin [Conference Presentation]. In: ASGCT 2021. (2021). 10.1371/journal.pone.0301175 PMC10994368 38574067

[238] Dembić Z Haas W Weiss S Mccubrey J Kiefer H Von Boehmer H. Transfer of Specificity by Murine α and β T-Cell Receptor Genes. Nature (1986) 320:232–8. doi: 10.1038/320232a0 2421164

